# qEEG and Functional Connectivity as a Translational Bridge Between Humans and Dogs in Epilepsy and Associated Disorders: From Spontaneous Model to Automatic Classification—An Integrative Review

**DOI:** 10.3390/vetsci13080803

**Published:** 2026-08-14

**Authors:** Dan Anghelinici, Mihai Musteata

**Affiliations:** Neurology Service, Faculty of Veterinary Medicine, Ion Ionescu de la Brad Iași University of Life Sciences (IULS), 700489 Iași, Romania; dan.anghelinici@iuls.ro

**Keywords:** quantitative EEG, qEEG, EEG coherence, functional connectivity, canine epilepsy, translational model, dog, canine cognitive dysfunction, integrative review

## Abstract

Quantitative electroencephalography (qEEG) converts the raw EEG signal into reproducible numerical descriptors (spectral power, hemispheric symmetry, coherence and signal complexity) and has emerged as a candidate translational biomarker linking human and canine neurology. This integrative review examined the diagnostic, prognostic, pharmacological and translational value of qEEG, with emphasis on functional connectivity, and assessed the comparability of the dog as a natural model of human disease. Seventy-five studies were included, spanning epilepsy, acute brain injury, neurodegeneration, rehabilitation and paroxysmal disorders. In both species, epilepsy was consistently associated with altered spectral power and with reduced or reorganized coherence, and interictal abnormalities were demonstrable even in the absence of visible epileptiform discharges. Dogs reproduced the human patterns closely: phenobarbital induced the spectral redistribution predicted by human pharmaco-EEG data, canine cognitive dysfunction reproduced the slowing and the sleep-architecture changes described in Alzheimer’s disease, and a single machine-learning pipeline classified human and canine recordings with comparable accuracy. Conversely, acute brain injury remains virtually unexplored in the dog, and no canine normative database comparable to the human ones is yet available. The evidence was limited by heterogeneous acquisition protocols, small samples and scarce longitudinal veterinary data. qEEG, and coherence in particular, appears to be a promising cross-species biomarker of network dysfunction and supports the dog as a translational platform, although standardized validation remains necessary.

## Simple Summary

Epilepsy and age-related brain disorders affect both people and dogs, and there is growing interest in using the dog as a natural model to understand these conditions. Quantitative EEG turns brain-wave recordings into numbers that can be compared across species. This review brings together human and canine studies to show where these quantitative brain-wave measures agree between people and dogs, and where more standardized research is still needed.

## 1. Introduction

Electroencephalography (EEG) is a fundamental non-invasive method for recording cortical electrical activity, extensively used in clinical practice and research to evaluate brain rhythms and paroxysmal phenomena generated by synchronized neural populations. From the first descriptions by Hans Berger in 1929 to the introduction of modern digital systems, the EEG has evolved from a technique based predominantly on visual interpretation into an analytical method capable of quantifying brain activity in real time [1,2].

The development of quantitative electroencephalography (qEEG) was made possible by the digitalization of the EEG signal and the application of advanced mathematical algorithms, including spectral analysis, measures of functional connectivity, entropy and complexity of Lempel–Ziv [2,3,4]. By converting the raw EEG signal into standardized numerical parameters (e.g., spectral power on frequency bands, hemispheric symmetry, inter- and intrahemispheric coherence, temporal complexity metrics), qEEG acquired the status of a validated and reproducible instrument, complementary to functional neuroimaging [4,5].

The current literature demonstrates the relevance of qEEG in the analysis of normal brain activity through its ability to quantify cognitive states and levels of consciousness. Recent studies have shown that non-linear measures, such as the complexity of Lempel–Ziv and spectral slope, can function as stable biomarkers of functional transitions between wakefulness and sleep, as well as changes induced by cognitive tasks [6,7]. qEEG has also been used to characterize cortical responses in complex physiological contexts, such as postural conditions [8], and for assessing diffuse cognitive dysfunction following systemic infections, including COVID-19 [9].

In brain pathology, qEEG has proven particularly useful in detecting network disorders that are difficult to identify by visual EEG. In epilepsy, various studies have documented alterations in spectral power, EEG coherence, and functional connectivity, even when there are no apparent epileptiform discharges [10,11,12,13]. However, the results are heterogeneous, and their interpretation is limited by methodological variations, the effects of antiepileptic medication, and the lack of standardization of qEEG protocols [3,4,14], this leads to uncertainties regarding the clinical and prognostic value of these parameters.

In epilepsy, qEEG provides an analytical framework that extends conventional visual EEG, allowing neural synchronization to be quantified and the reorganization of the cortical networks involved in pathogenesis to be evaluated [4,13,15]. Studies have shown that connectivity measures, in particular EEG coherence, can have predictive value for therapeutic response, risk of seizure recurrence and post-surgical prognosis [15,16]. In addition, qEEG is a robust tool for neuropharmacological studies, facilitating objective evaluation of the effects of antiepileptic drugs on brain networks [17,18,19].

In veterinary medicine, interest in qEEG is steadily growing, especially in the study of canine epilepsy and sleep [20,21,22]. Recent studies have standardized non-invasive EEG protocols in dogs and have shown significant similarities between canine and human epileptiform models [20,23]. These data support the use of the dog as a natural model of spontaneous epilepsy with translational value for human epileptology [24].

Recent reviews have addressed non-invasive EEG in dogs [20] and the electrode configurations available for canine recordings [23], and the dog has been proposed as a natural model of epilepsy on largely qualitative grounds [24]. These contributions were nevertheless confined either to technical aspects or to a single disorder or species. To our knowledge, no synthesis has yet covered the broad spectrum of neurological conditions in which qEEG is applied while systematically examining functional connectivity measures as candidate translational biomarkers in the comparative human–dog context. The present review is therefore not an update or a replication of an existing systematic review, but a synthesis that integrates human and veterinary evidence within a single comparative and translational framework, covering multiple neurological domains and focusing on the added value of qEEG over conventional visual analysis.

**Figure 1:**
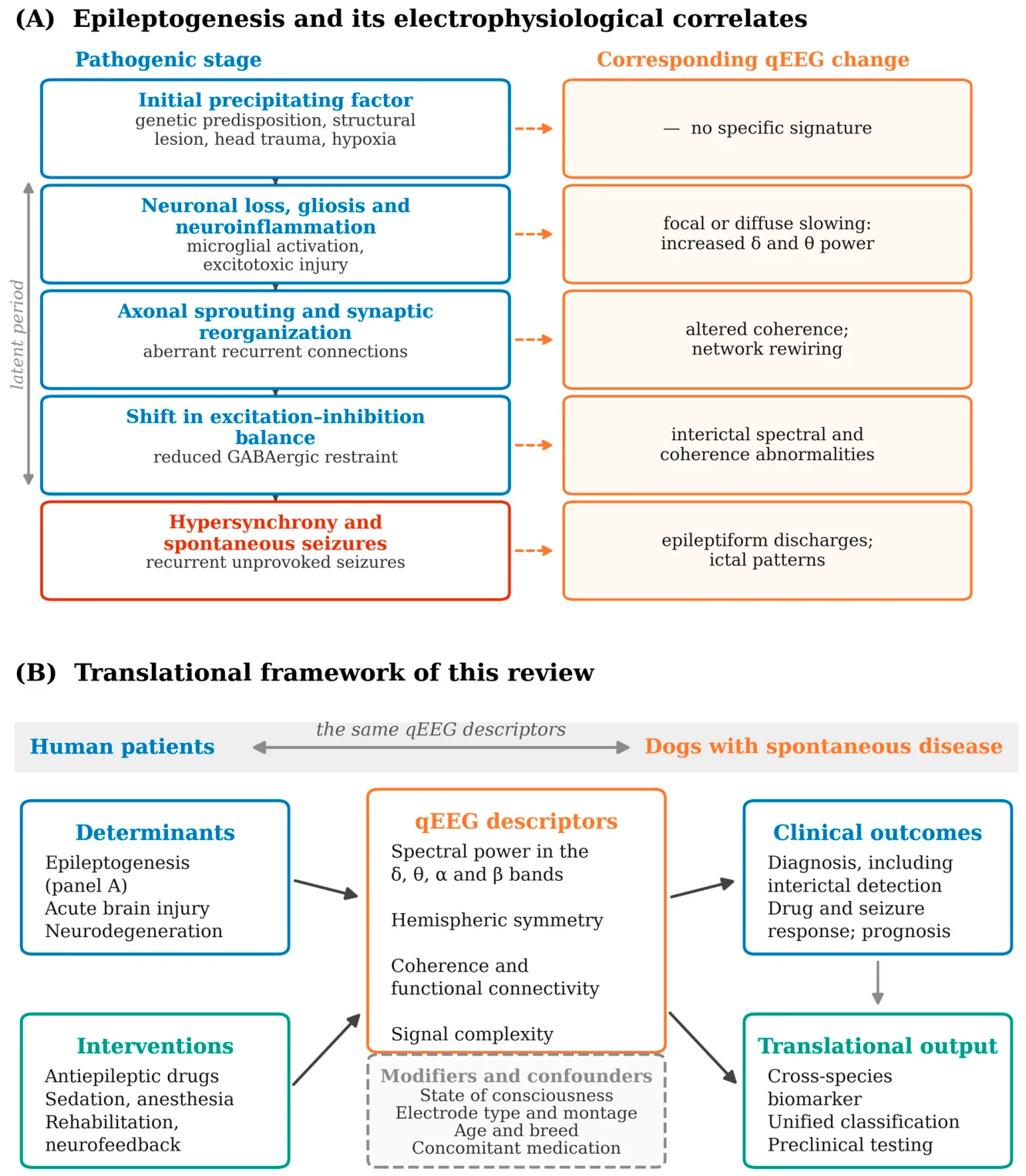
Pathogenesis of epilepsy and the translational framework of this review. (**A**) Successive stages of epileptogenesis, from the initial precipitating factor through neuronal loss, gliosis and neuroinflammation, axonal sprouting and synaptic reorganization, and the resulting shift in the balance between excitation and inhibition, to hypersynchrony and spontaneous recurrent seizures. The interval between the initial insult and the first spontaneous seizure constitutes the latent period, the window within which an antiepileptogenic intervention would have to act. The right-hand column gives the qEEG change that corresponds to each stage; note that the earliest stage has no specific electrophysiological signature, which is why qEEG cannot at present identify an animal or patient before the pathological process is established. (**B**) The same qEEG descriptors are applied in human patients and in dogs with spontaneous disease, which is what makes the cross-species comparison possible. The dashed box lists the factors that must be controlled or reported before any such comparison is interpretable; these are specified in Section 6. The scheme is drawn from the evidence reviewed in Section 3, Section 4, Section 5, Section 6, Section 7 and Section 8 and is intended as an orientation to the argument rather than as a complete account of epileptogenesis.

Moreover, qEEG allows the investigation of the mechanisms by which therapeutic interventions, especially antiepileptic medication, influence brain activity [18,19]. Antiepileptic drugs can modulate cortical excitability, neural synchronization and functional connectivity, effects that are reflected in changes in spectral power and EEG coherence [18,19,25,26,27]. These changes may differ between species, depending on pharmacodynamic peculiarities and the organization of neural networks [21,25,28,29], emphasizing the importance of comparative approaches. The application of qEEG in epilepsy is characterized by a high degree of complexity, determined by the state of consciousness (wake, sleep, sedation), the effects of pharmacological treatment, interspecies anatomical differences and variability of EEG recording protocols [4,20,30,31]. The stages through which an initial insult gives rise to spontaneous seizures, and the qEEG change that corresponds to each of them, are summarized in Figure 1A. Figure 1B places this cascade within the wider framework of the present review, relating the determinants of network dysfunction and the interventions applied to it (antiepileptic medication, sedation, rehabilitation) to the qEEG descriptors through which both are measured, and to the clinical and translational outcomes against which those descriptors are validated.

The main objective of this review is to synthesize and critically evaluate the existing evidence on the use of quantitative electroencephalography (qEEG), with a focus on functional connectivity measures, in particular EEG coherence, in epilepsy, in both human and veterinary medicine. To this end, the human and veterinary literature was synthesized using an integrative-review approach [32], a method particularly suited to combining heterogeneous experimental, observational and methodological sources into a unified comparative framework.

Accordingly, the review was organized around the gaps identified above. Because the qEEG changes reported in human epilepsy and in canine idiopathic epilepsy have not previously been placed side by side using the same descriptors, they were first described and compared directly, in order to establish how far connectivity measures behave as functional biomarkers of the network dysfunction that underlies the disease in either species. Since therapeutic effects on qEEG have been characterized almost exclusively in human patients, the review then examined how antiepileptic medication and other interventions are reflected in the organization and dynamics of neural networks, and whether the drug-specific spectral signatures described in humans recur in the dog. Finally, because a translational argument rests on similarity but is most informative where similarity fails, interspecies convergence and divergence were addressed explicitly in order to weigh the dog as a natural model of epilepsy, and the methodological limitations of the available evidence were identified so that the standardization and clinical validation still required could be specified.

## 2. Materials and Methods

This work was conducted as an integrative review [32]—a method that permits the combined synthesis of experimental, observational, methodological and theoretical studies addressing a common question. It followed the successive stages of problem identification, systematic literature search, evaluation of the retrieved studies and narrative data analysis. Titles and abstracts, and subsequently full texts, were assessed against the eligibility criteria by the review team, with disagreements resolved by discussion, and the retained studies were tabulated in a standardized evidence table (Table S1). Given the methodological heterogeneity of the included studies, no meta-analysis was performed and no formal risk-of-bias instrument was applied; the synthesis is qualitative and comparative. Although an integrative review does not require the PRISMA checklist, which was developed for systematic reviews and meta-analyses [33], the reporting of the search, of the eligibility criteria and of the selection process followed PRISMA 2020 principles wherever these were applicable to a narrative synthesis.

### 2.1. Search Strategy

A review of the literature on the use of quantitative electroencephalography (qEEG) as a functional and translational marker in epilepsy has been carried out, with a focus on comparing humans and dogs. The PubMed, Web of Science, Scopus and Google Scholar databases were queried using keywords such as: (qEEG OR quantitative EEG OR quantitative electroencephalography) AND (epilepsy OR seizures OR sleep) AND (human OR people OR canine OR dog OR veterinary) AND/OR (biomarker OR functional marker OR translational model OR treatment response OR antiepileptic drugs). The search covered articles published between 1994 and January 2026, with no language restrictions.

### 2.2. Inclusion and Exclusion Criteria

Original studies were included regardless of design (experimental, randomized/nonrandomized, observational, case series, case reports), which investigated qEEG in human subjects or dogs (with epilepsy, healthy or other intracranial pathologies). Both studies using qEEG as the main or complementary method (spectral analysis, frequency bands, coherence, functional connectivity) and those using only conventional EEG were accepted, provided that they provided relevant data for translational comparability.

Articles without original EEG data (editorials, letters to the editor, narrative reviews, expert opinions) and studies that did not provide any EEG parameters were excluded. The same rule was applied for reports that did not clearly specify the methodology for recording and/or analysis, as well as studies conducted on species other than human and dog (unless they provided reference data essential for comparative understanding). On the same grounds, a small number of feline and canine–feline studies were retained where they provided methodological data essential for cross-species comparability, in particular the validation of unsedated recording protocols applicable to both species. Finally, reports that did not provide access to the full text or sufficient data to extract information according to the standardized form were excluded.

### 2.3. Study Selection

**Figure 2:**
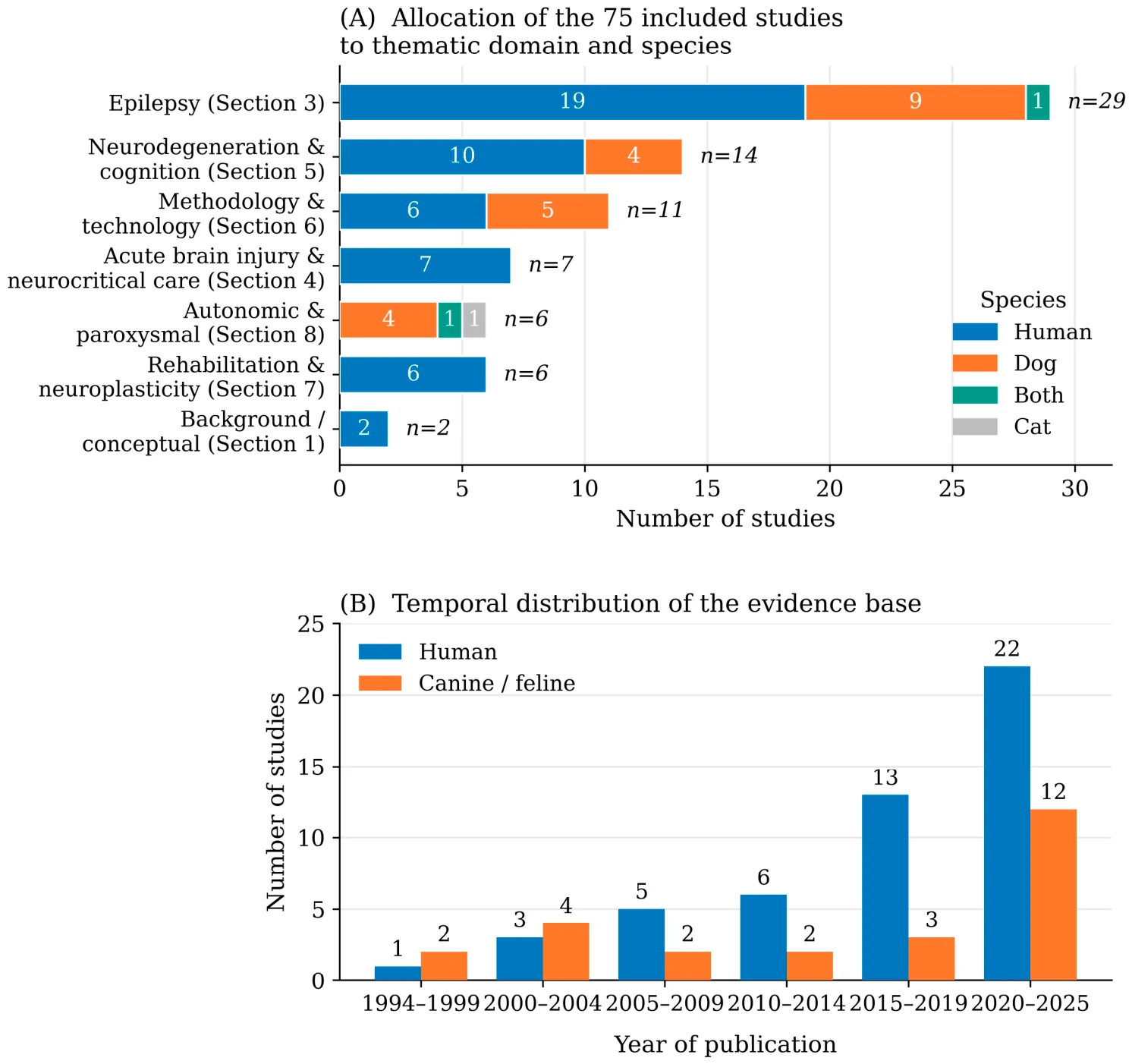
Constitution of the evidence base of this integrative review. (**A**) Allocation of the 75 included studies to the six thematic domains of the synthesis and to species. Each study was assigned to the domain corresponding to the section in which it is discussed; where a study is cited in more than one section (28/75), it was assigned to the section in which it is cited most frequently. Seven studies that inform the conceptual framework only are shown separately. (**B**) Year of publication of the included studies, grouped in five-year intervals. Counts are derived from Table S1. Species categories: human; dog; both species within the same study; cat.

The selection was made in two stages. In stage 1, accessible studies (title, summary) that met the type of study criterion were selected. In stage 2, by the analysis of the full text, the criteria regarding population, method and results were applied, and the data were recorded in an Excel table. The number of records retained at each stage, together with their allocation to the six thematic domains and to the two species, is summarized in Figure 2.

### 2.4. Data Collection and Data Items

The data were extracted with a standardized form, centralizing the following information according to the table structure: Author (Year); Species; Condition/Model; Study design; Number of subjects; EEG/qEEG Objective; Model /EEG configuration; Anesthesia; Main results; EEG (classical) Pattern; qEEG Pattern; Clinical /behavioral Correlates; and Translational relevance.

The full evidence table is provided in the Supplementary Materials (Table S1).

The studies were analyzed descriptively according to design (randomized, observational, series of cases, etc.) and methodological characteristics, without formal ranking of the level of evidence. The quality assessment considered factors such as blindness, randomization, control groups, sample size, and duration of the study.

A standardized bias risk assessment tool was not applied; however, a narrative analysis of the clarity of criteria, sample size, protocol description, control of confounding factors and reporting consistency was carried out. No meta-analysis was performed and the measures of effect were not summarized quantitatively due to methodological heterogeneity and variability of qEEG parameters.

Given the significant methodological and clinical heterogeneity of the studies included, the results were summarized through a narrative and comparative approach without a statistical meta-analysis. The synthesis was structured on major thematic groups, as follows:

1. Epilepsy—modeling, diagnosis and management with qEEG;

2. Acute brain injury and neurocritical care (TBI, stroke, hemorrhage);

3. Neurodegeneration and cognitive dysfunction (CCD/Alzheimer’s model);

4. Methodology and technology bridge (qEEG standards and analysis);

5. Rehabilitation and neuroplasticity;

6. Autonomic and paroxysmal disorders.

The narrative approach enabled comparative integration of human and veterinary studies, highlighting recurrent patterns of qEEG changes; specific species differences; and translational relevance for neurological and clinical research. A quantitative synthesis or statistical evaluation of heterogeneity was not conducted; the consistency of the results was assessed narratively, based on the consistency of the trends reported between similar studies.

## 3. Epilepsy—Modeling, Diagnosis and Management with qEEG

Epilepsy is the most well-explored field in terms of translational qEEG application, both because of its high prevalence in both species, and because of the remarkable similarities between human and canine electrophysiological patterns. In human epilepsy this position rests on a consistent quantitative signal: spectral abnormalities are demonstrable in temporal-lobe epilepsy even when the recording is visually normal [13], resting-state spectral power is altered in patients with epilepsy and psychiatric comorbidity [10], and inter- and intrahemispheric coherence discriminates epileptic from nonepileptic activity [15]. These observations define the descriptors against which the canine data reviewed below may be read. In this section, we discuss how fundamental research on spontaneous canine epilepsy has strengthened the dog’s position as a natural model for human epilepsy, the manner in which the background qEEG has become an objective biomarker independent of the presence of epileptiform discharges. Furthermore, we analyze how the pharmacodynamic response to antiepileptic treatment can be quantified by spectral changes, paving the way for objective monitoring of therapy in both species.

### 3.1. Spontaneous Canine Epilepsy as a Natural Model for Human Epilepsy

Over the past two decades, translational research in epilepsy has benefited greatly from the use of the dog with spontaneous epilepsy as a natural model. The first decisive step in this direction was taken by Berendt et al. [34], who demonstrated a remarkable similarity between the electroencephalographic patterns of dogs with idiopathic epilepsy and those found in human focal epilepsy, particularly the temporal lobe form. The anomalies described—spikes, sharp waves, focal slow waves and regional patterns—show almost-perfect morphological correspondence with those in human neurological practice. The authors concluded that canine epilepsy constitutes a robust natural model for humans, which justifies the integration of this species into the circuits of translational research.

Building on this basis, Jeserevics et al. [35] introduced an innovative paradigm by using quantitative electroencephalography (qEEG) for benchmarking of healthy dogs and Finnish Spitz diagnosed with epilepsy. The study found that the quantitative analysis of the EEG background activity reveals consistent differences in spectral power distribution and prevalence of epileptiform activity, sufficient to statistically discriminate between the two groups. The essential contribution of this approach is the proposal of the qEEG of the background activity as an objective biomarker for canine epilepsy, thus transcending the traditional role of the EEG, which had hitherto been limited to morphological confirmation of paroxysmal discharges. A natural extension of this approach was carried out by Bocheńska et al. [36], who investigated the effects of phenobarbital treatment in dogs with idiopathic epilepsy, in a longitudinal pre–post-intervention design. qEEG analysis showed significant changes in spectral profile under therapy, a reduction in delta band power and a redistribution of balance between slow and fast bands, changes that were correlated with clinical seizure control. The authors interpreted these patterns as potential pharmacodynamic markers of phenobarbital response, emphasizing their direct translational applicability in epilepsy management.

Overall, the three studies illustrate a coherent methodological development path: qualitative validation of the natural canine model [34], transition to quantitative biomarkers derived from background activity [35] and their use for objective monitoring of therapeutic response [36]. The common elements that emerge are: (i) recognizing the dog as an exceptional translational model for human epilepsy; (ii) adopting qEEG as a tool for detecting subtle but clinically relevant changes; and (iii) the potential of these parameters to become useful pharmacodynamic markers in current practice. This synthesis demonstrates that the integration of the dog into the translational research circuits is not only justified, but has already generated quantitative tools with direct applicability in the development of new therapies and in the optimization of epilepsy management, both in veterinary medicine and in human medicine. These developments build on the earliest systematic canine recordings by Pellegrino and Sica [37], who described the canine EEG technique and its interictal findings in normal and epileptic dogs, and are complemented by Wrzosek et al. [38], who related epileptiform discharges to background activity across dogs with seizures of differing etiologies, reinforcing the diagnostic value of background qEEG independently of overt paroxysmal discharges.

### 3.2. Experimental Models and Comorbid Conditions

A key direction in strengthening translational research with canine qEEG is the understanding and control of variables that can alter the interpretation of quantitative parameters. This category includes studies that address the effects of sedation, differential diagnosis of nonepileptic paroxysmal events, and the development of standardized experimental models of convulsive provocation.

A fundamental methodological contribution belongs to Itamoto et al. [30], who investigated the dose-dependent effects of medetomidine sedation on canine EEG. The results demonstrate a marked and dose-proportional slowdown in EEG activity, materialized by increased slow activity. This finding has crucial implications for the design of qEEG studies in dogs as it requires the interpretation of quantitative parameters in direct relation to the anesthetic or sedation protocol used. Without such detailed reporting, comparability between studies and translational applicability of results become compromised. The same group subsequently extended this work to sedative combinations, showing that medetomidine–midazolam and medetomidine–midazolam–butorphanol each produce a distinct quantitative EEG profile [39], so that the agent combination, and not merely the presence of sedation, has to be specified when canine qEEG data are reported.

On another plane, but with the same relevance for the accuracy of the diagnosis, Bush et al. [40] reported a case of REM sleep behavior disorder (RBD) in a dog with epilepsy and associated neurodegenerative conditions. Polysomnographic EEG monitoring confirmed that complex behavioral episodes occur consistently during REM sleep, presenting specific patterns in the absence of epileptiform activity. This single case is consistent with canine RBD as a potential spontaneous model for the corresponding human pathology and emphasizes the usefulness of polysomnographic EEG recording in the differential diagnosis of nocturnal seizures versus parasomnias. In translational practice, this distinction is essential to avoid overdiagnosis of epilepsy and the correct selection of subjects in clinical trials.

Completing this methodological perspective, Bassett et al. [41] developed a standardized convulsive challenge model on the beagle, using picrotoxin and pentylenetetrazole (PTZ) in the context of safety pharmacology. The model produces replicable seizures with controllable duration and severity, allowing precise correlation between the administered dose and the EEG and behavioral response. This tool is essential both for screening the pro-seizure potential of new molecules in the preclinical phase, and for calibration and validation of the EEG quantitative analysis algorithms. By providing a standardized framework, it facilitates the development of objective qEEG metrics, which can then be applied to spontaneous models of epilepsy.

Overall, the three studies illustrate a complementary direction to translational research based on spontaneous epilepsy: they focus on the confounding variables and methodological tools needed to ensure the robustness and reproducibility of qEEG data. Whether in sedation control, differential diagnosis of parasomnias or the use of standardized challenge models, these contributions strengthen the foundation on which the application of qEEG in comparative human–dog studies is based. A further caveat with direct clinical relevance was raised by Ukai et al. [42], who compared objective, EEG-derived counts of ictal paroxysmal discharges with owner-reported seizure frequency and documented substantial under-reporting in canine epilepsy—a phenomenon well recognized in human epileptology. This finding suggests that objective electrographic quantification may be necessary for reliable seizure ascertainment in both species, and cautions against relying on caregiver histories as the sole outcome measure in translational studies.

### 3.3. Methodological Consistency, Connectivity and Standardization

A natural extension of conventional spectral analysis is the investigation of brain functional connectivity through EEG coherence, a paradigm that allows the transition from local activity description to understanding the integration of neural networks. In this context, Preobrazhenskaya [43] conducted an experimental longitudinal study focused on intrahemispheric EEG coherence in dogs, aiming to identify regional patterns that reflect the functional organization of the canine cortex. The study has demonstrated that these patterns of coherence change under pathological conditions, thus providing an interpretative framework for connectivity measures comparable to those used in human studies. The contribution of this approach is to validate that metrics such as the coherence of the EEG, widely used in human neurology for mapping dysfunctional networks in epilepsy, can be applied similarly to the dog, thus strengthening the common basis for translational research. It should be noted, however, that the canine coherence evidence remains scarce: to date it rests essentially on a conditioning study [43] and on a methodological comparison of electrode types [44], with no study having yet characterized EEG coherence in canine epilepsy itself. At this stage, coherence is therefore best regarded as a promising but still largely unvalidated cross-species marker in the dog.

However, the translational potential of connectivity measures can only be achieved under strict methodological rigor. Musteata et al. [44] compared, within the same subjects, different types of electrodes (emphasis on stainless steel-needle-type electrodes) in canine EEG recordings. The main result shows that the type of electrode significantly influences the coherence measures and other quantitative parameters of qEEG. This finding has profound implications for the domain: even when recording protocols are apparently similar, electrode-type differences can introduce variations that mask or distort real biological differences. The authors conclude that recording instrumentation standardization is a sine qua non for the validity of comparative and translational studies, both between laboratories and between species.

Overall, the two studies illustrate an essential dichotomy in the development of translational qEEG: on the one hand, it is necessary to develop metrics with profound neurobiological significance (such as EEG coherence) that capture the functional organization of the brain and its dysfunctions in epilepsy; on the other hand, the translational applicability of these metrics critically depends on the rigorous standardization of the acquisition equipment. Without this double anchoring, biological and methodological comparability between human and canine studies remain compromised. The works of Preobrazhenskaya [43] and Musteata et al. [44] therefore provide both a conceptual direction and a methodological warning for future research in the field.

### 3.4. Cross-Species Methodological Integration Through Machine Learning

The methodological path described in previous sections (from the qualitative validation of the canine model, to the development of quantitative biomarkers, the rigorous control of confounding variables and the exploration of functional connectivity) points toward a fundamental concern about the possibility of unifying the EEG analysis across species in a common computational framework. Levitt and collaborators [45] propose a unified cross-species analysis platform, based on the use of the same Support Vector Machine (SVM) algorithm (a supervised machine learning method) trained on a narrow set of statistical features extracted from the EEG signal. The results obtained are remarkable from a translational perspective: the algorithm achieved comparable performance in distinguishing artifact-contaminated from clean EEG segments in both humans and dogs, indicating that a single preprocessing and classification pipeline can operate with similar accuracy across species. It should be emphasized, however, that this proof of concept concerns automated artifact detection rather than the classification of disease states, so its translational scope is, at present, primarily methodological. By providing a common analytical pipeline, the study suggests that the dog may be approached not merely as a clinical analog but as a component of a shared computational framework, in principle enabling the joint analysis of data from both species. This remains, for the moment, a methodological demonstration, and its extension from artifact detection to the classification of disease states will require dedicated validation. This latter step has begun to be addressed directly: Wang et al. [46], in a study emblematically entitled “Canine EEG helps human”, used deep-learning domain adaptation and knowledge distillation to align canine and human EEG and achieved cross-species and cross-modality seizure detection with areas under the curve exceeding 0.90, even with very limited labeled data in the target species. Crucially, knowledge could be transferred in both directions—from dog to human and from human to dog—providing the strongest evidence to date that canine data can materially improve human seizure-detection models, and thereby extending the unified computational framework beyond artifact handling to the classification of disease states themselves. This contribution has profound implications for the future of translational research in epilepsy. First, it paves the way for the development of diagnostic and monitoring tools that can be applied directly to both human and veterinary medicine without species-specific adaptations. Secondly, by allowing for the common aggregation and analysis of data from both sources, the proposed SVM platform can contribute to significantly increasing the statistical power of studies, thus offsetting the limitations imposed by the often-small sample sizes in spontaneous model research.

Finally, the study of Levitt et al. [45] validates that the methodological efforts described in the previous sections (e.g., standardization of protocols, control of confounding variables, development of robust metrics) have created the necessary premises for an authentic computational integration between species, transforming translational research from a field of descriptive analogies to one of unified predictive models.

### 3.5. Contributions from Human Literature Relevant to the Canine Model

#### 3.5.1. Interictal and Pharmacodynamic Patterns of qEEG in Human Epilepsy

Validation of the dog as a translational model in epilepsy requires not only the demonstration of phenotypic similarities between species, but also the alignment of canine qEEG metrics to interpretative frameworks already established in human neurology. The human literature provides both the conceptual basis for quantitative biomarkers and essential methodological benchmarks for the design of translational studies. A fundamental contribution in this direction belongs to Drake et al. [11], who investigated the manifestations of qEEG in the interictal period of human epilepsy. The study demonstrated persistent anomalies between seizures (e.g., relative power changes in frequency bands and focal slowdown patterns) even in the absence of visible epileptiform discharges on conventional EEGs. These “interictal” qEEG signatures constitute sensitive biomarkers of epileptic reactivity, providing a subtle diagnostic window rather than simply detecting the paroxysmal activity. For translational research, this finding has direct relevance: it validates the use of the same quantitative metrics to characterize interictal activity in the dog, thus extending beyond the traditional pattern of visual epileptiform discharges. Consistent with this, more recent human studies have broadened the interictal qEEG repertoire: Fonseca et al. [47] reported reproducible interictal spectral changes in temporal-lobe epilepsy even on visually unremarkable recordings; Varatharajah et al. [48] showed that quantitative analysis of visually normal scalp EEG could predict seizure freedom after anterior temporal lobectomy; and Segovia-Oropeza et al. [49] found that increased delta–theta power and altered theta connectivity after a first unprovoked seizure were associated with subsequent epilepsy. Together, these findings indicate that interictal qEEG and connectivity may carry diagnostic and prognostic information beyond visible discharges—an inference directly transferable to the design of canine studies. Further work has linked connectivity and treatment response: Hwang et al. [50] found that increased EEG coherence predicted medical refractoriness in temporal-lobe epilepsy managed on monotherapy; Zahnert et al. [51] showed that quantitative EEG markers predicted the outcome after the initial treatment with levetiracetam in newly diagnosed epilepsy; and Yan et al. [52] derived epileptogenicity biomarkers with early-prognostic value from stereo-EEG in drug-resistant cases. That EEG coherence in particular should emerge as a predictor of pharmacoresistance is directly pertinent to the present review, since coherence is precisely the measure proposed here as the human–dog translational bridge.

Clemens et al. [53] made an essential contribution by analyzing adult and pediatric patients with epilepsy under various therapeutic protocols including carbamazepine, valproic acid and others. The study identified drug-specific qEEG profiles materialized in changes in mean frequency and spectral power distribution. These “specific pharmaco-footprints” have a double significance: (i) clinically, they can serve as markers of treatment compliance or therapeutic response; (ii) translationally, they provide a reference framework for interpreting data obtained in dog EEG pharmacological studies, allowing direct comparison of spectral effects of the same molecules between species.

Beyond epilepsy itself, the human literature documents the wider applicability of qEEG in acute neurological pathology, with direct implications for veterinary medicine. Claassen and collab. Ref. [54] demonstrated the potential of qEEG as a continuous monitoring tool in intensive neurological therapy, identifying that the power reduction in a determined frequency band was a superior predictor of adverse evolution in patients with severe subarachnoid hemorrhage. The principle applied (using a simple quantitative parameter for real-time prognosis) is directly transposable in veterinary intensive care units, where objective monitoring of critical neurological patients remains a major challenge.

Extending this logic of quantitative decision support, Ayaz et al. [55] proposed a discriminating qEEG (score with the threshold set at ≥31) for identifying patients with mild-to-moderate brain trauma requiring additional imaging investigations and intensive monitoring. The study demonstrates the ability of qEEG to partially complement subjective evaluation by providing greater sensitivity to clinically significant lesions. For veterinary medicine, this approach opens the way for the development of analog triage tools, allowing more efficient allocation of diagnostic resources and early identification of high-risk canine patients. Overall, human studies provide a key conceptual and methodological framework for translational research using the canine model by validating the existence of interictal qEEG biomarkers, identifying drug-specific pharmacodynamic landmarks, demonstrating the applicability of continuous quantitative monitoring in acute pathology, and introducing the principle of qEEG-based decision-making scores. All these contributions are directly relevant to the design and interpretation of canine studies, ensuring that the metrics developed in the dog are aligned with the standards and interpretative frameworks already established in human neurology.

#### 3.5.2. Normative Data, Variability and Theoretical Frameworks

The shift from exploratory use of qEEG to its application as a valid clinical tool requires three fundamental categories of knowledge: understanding the distribution of parameters in healthy populations, having robust theoretical frameworks for interpreting connectivity metrics, and demonstrating its usefulness in guiding therapeutic interventions. The human literature provides, in these areas, essential landmarks that are directly applicable in the context of the canine model.

A fundamental contribution in the first of these directions belongs to Van Albada et al. [56], who provided a comprehensive set of normative data for human qEEG parameters, including a detailed analysis of intra and inter-individual variability in healthy populations. These benchmarks are essential for the objective definition of pathological deviations at the level of spectral power, frequency and coherence, allowing the calibration of the abnormality thresholds on solid statistical bases, not on subjective qualitative assessments. For translational research, this contribution is of deep relevance: it stresses that the development of normative datasets for dogs (adapted to the peculiarities of breed, age and registration conditions) is a prerequisite for the validation of any qEEG biomarker in veterinary medicine.

On the interpretative plane, Srinivasan et al. [57] developed a methodological conceptual framework for understanding the coherence of EEG and MEG as a measure of functional connectivity. Their essential contribution lies in the explanation of the relationship between cortical sources, activity generated at the neuronal level, and the network patterns observed at the scalp. This theoretical model is of paramount importance in order to avoid reductionist or misleading interpretations of coherence measures, which may be influenced by factors such as conductive volume, common reference or recording noise. Directly, this framework is also applicable in the context of canine coherence studies, providing a unitary basis for interpreting connectivity metrics in both humans and dogs. He assures readers that when researchers compare patterns of coherence between species, they speak the same neurophysiological language. Extending this paradigm from interpretation to action, Walker [58] has documented the clinical applicability of qEEG in guiding neurofeedback therapy in patients with epilepsy and comorbid disorders. The study demonstrated that quantitatively guided approaches—therapeutic approaches in which qEEG parameters are used to customize the neurofeedback protocol—are associated with superior results compared to non-specific interventions. This finding has a profound significance: qEEG thus transcends its traditional role, which was limited to diagnosis and characterization, becoming an active tool of therapeutic guidance. For veterinary medicine, this direction opens up new insights, including the possibility of developing personalized neurofeedback protocols for epileptic dogs, tailored on the basis of their individual qEEG profiles.

Overall, the three studies—Van Albada et al. [56], Srinivasan et al. [57] and Walker [58]—provide a conceptual continuum that transforms qEEG from a measuring technique into a complete clinical tool. The first provides the statistical foundation (regulatory data), the second provides the interpretative framework (connectivity theory), and the third demonstrates the value added in the therapeutic plan (intervention guideline). For translational research with the canine model, these contributions are essential: they define the methodological benchmarks that canine studies must follow to achieve the same level of clinical rigor and applicability, and validate the directions in which canine qEEG can evolve—from an exploratory research tool to a diagnostic tool, prognosis and therapeutic guide in veterinary medicine.

#### 3.5.3. Extensions to Other Neurological and Psychiatric Pathologies

Although the focus of this review is epilepsy, the human literature documents a much wider applicability of qEEG, covering a broad spectrum of neurological and psychiatric pathologies. These extensions are of paramount importance for translational research as they demonstrate that the canine model originally validated in epilepsy can be successfully extended to other areas, from neurodegeneration to psychotic disorders and from behavioral disorders to the effects of aging.

A particularly promising extension direction is neurodegeneration, particularly Alzheimer’s disease and its canine counterpart, cognitive dysfunction syndrome. Garn et al. [59] identified that dependent qEEG biomarkers differentiated between open eye conditions, closed eyes and cognitive performance in patients with early to moderate Alzheimer’s disease. These markers, which combine spectral parameters with coherence measures, are of direct relevance to the study of canine cognitive dysfunction, thus extending the applicability of the model beyond epilepsy to the field of neurodegeneration. Since the dog spontaneously develops a cognitive dysfunction syndrome with remarkable neuropathological and behavioral similarities to human Alzheimer’s disease, these qEEG biomarkers provide objective tools essential for early diagnosis and monitoring of disease progression in both species. A complementary contribution, with profound methodological implications, comes from Kaplan et al. [60], who investigated the correlations between polysomnographic EEG parameters and cognitive status. The study revealed a significant dissociation between objective EEG measurements and subjective perception of cognitive status—an observation with important methodological implications for the interpretation of behavior in nonsedated animals. This finding underlines a fundamental limitation of purely behavioral assessments in translational studies: subjective perception (either of the patient or the observer) may not faithfully reflect the neurophysiological reality. For canine research, it validates the use of qEEG as an objective alternative to behavioral interpretations that may be affected by the observer’s bias or the difficulty of assessing subjective states in the animal.

The ability of qEEG to discriminate against phenotypic subtypes within the same diagnostic category has been demonstrated by Youh et al. [61]. The study identified differential patterns of connectivity and coherence—in particular changes in the alpha-interhemispheric coherence—between patients with pure major depression and those with comorbid major depression with video game addiction. This finding has profound relevance for translational research: it demonstrates that qEEG can provide objective stratification of patients, exceeding the limits of clinical diagnostic categories that can aggregate pathophysiologically distinct entities. In the context of canine epilepsy, where there is a significant heterogeneity of treatment response and progression, the application of the same principle could allow the identification of phenotypic subtypes with different prognosis, thus guiding personalized therapeutic strategies.

Naim-Feil et al. [62] made an essential contribution by investigating the dynamics of the EEG network in patients with schizophrenia during a cognitive task. The study revealed time-sensitive dysfunctions of connectivity, i.e., alterations in the way connectivity changes during the task, attesting to the sensitivity of dynamic network measures to psychotic disorders. This approach goes beyond the limits of static connectivity analyses, capturing subtle aspects of neural dysfunction that may not be apparent at rest. For translational research, the paradigm of cognitive tasks can be adapted to the dog, allowing assessment of network dynamics in canine models of behavioral or cognitive disorders, and thus providing a more nuanced window on the pathophysiology of these conditions. The applicability of qEEG to anxiety disorders was further illustrated by Kopańska et al. [63], who reported characteristic amplitude patterns (a low contribution of the sensorimotor rhythm together with elevated high-beta activity) in patients with generalized anxiety disorder, adding anxiety to the widening range of conditions in which quantitative signatures can be defined and, potentially, modeled in the dog. Individual-difference research points in the same direction: Wacker [64] related extraversion, dopaminergic function and positive emotion to frontal EEG asymmetry and to cognitive stability and flexibility, illustrating that qEEG can index temperament-linked traits—a dimension of potential relevance for the behavioral phenotyping of dogs.

Strengthening the evidence for qEEG as a marker of therapeutic response, Fogelson et al. [65] reported post-treatment qEEG changes in an open-label longitudinal study, characterized by increased alpha power and reduced delta and theta activity. These changes were correlated with clinical improvement, thereby strengthening the validity of qEEG as an objective monitoring tool for the effectiveness of therapeutic interventions. This direction aligns perfectly with the pharmaco-EEG studies in canines [36], providing a reference framework for the interpretation of spectral changes induced by antiepileptic treatment.

Finally, Terry et al. [66] and Cao et al. [67] contributed essential data on modulating factors of qEEG patterns—the influence of aging, sleep stages and epileptic phases. These milestones are of paramount importance for studies in dogs at different stages of the disease, as they define the range of normal physiological variation within which pathological deviations should be interpreted. Without a detailed knowledge of how age, sleep/wake state and epileptic cycle phase influence qEEG parameters, the likelihood of confusion between the effects of pathology and normal physiological variations remains high.

Overall, the six contributions presented significantly expand the applicability framework of qEEG and the canine model. They demonstrate that the quantitative tools developed in the context of epilepsy are applicable in a wide range of pathologies—from neurodegeneration to psychotic disorders and from behavioral disorders to the effects of aging—and that the methodological principles set out in the human literature provide essential landmarks for canine studies. This extension reinforces the translational value of the canine model, transforming it from an instrument exclusively dedicated to epilepsy into a general platform for comparative neurological and psychiatric research.

### 3.6. Translational Human–Dog Model

The validation of the dog as a natural model of human epilepsy is based on a convergence of the literature over the last two decades, which demonstrates phenotypic, electrophysiological and therapeutic response similarities between the two species. This integrative synthesis aims to articulate the three main levels of translational correspondence (clinical, quantitative and computational) and to highlight their implications for future research.

#### 3.6.1. Clinical and Phenotypic Correspondence

The literature converges on the validation of canine idiopathic epilepsy as a natural model of human epilepsy, based on remarkably similar EEG patterns. Berendt et al. [34] demonstrated that spike-type discharges, focal slow waves and regional patterns recorded in dogs with spontaneous epilepsy are almost morphologically identical to those encountered in human focal epilepsy, particularly the temporal lobe form. This observation was extended by Jeserevics et al. [35] by introducing quantitative analysis, and Bocheńska et al. [36] showed that phenobarbital-induced spectral changes correlate with clinical seizure control, providing a direct pharmacodynamic model comparable to human.

In parallel, studies on human patients—such as Drake and collaborators [11] on the interictal manifestations, Clemens et al., [53] on the qEEG profiles specific to the different antiepileptics drugs and Walker [58] on guided neurofeedback—provided a repertoire of qEEG signatures that can be prospectively validated in the dog. This double direction—from man to dog and from dog to man—strengthens translational correspondence at the phenotypic level.

#### 3.6.2. Quantitative Biomarkers and Connectivity Metrics

The comparative analysis of the literature reveals a common set of translational qEEG parameters, organized into three main categories.

The first category is spectral power in frequency bands. This parameter is used extensively in both human epilepsy [11] and in the canine [35,36], but also in neurodegenerative pathologies [59] and in traumatic brain injury [55], which gives it remarkable versatility. In dogs, for example, the increase in delta and theta power in the background activity was correlated with the severity of epilepsy, while in humans, the same parameters are used to monitor the response to treatment.

The second category includes coherence and network metrics. A theoretical framework developed in the human context by Srinivasan et al. [57] enabled the correct interpretation of EEG coherence as a measure of functional connectivity, considering phenomena such as volumetric conduction and common sources. These principles were applied experimentally to the dog by Preobrazhenskaya [43], who identified regional patterns of intrahemispheric coherence, and Musteata et al. [44], who showed that the electrode type significantly influences connectivity measures—a key finding for translational standardization.

The third category includes discriminatory scores and compositions. Ayaz et al. [55] proposed a qEEG score with a ≥31 threshold for triage of mild traumatic brain injury in humans, and Youh et al. [61] and Naim-Feil et al. [62] used similar metrics to discriminate against psychiatric subtypes. These approaches have a direct potential for replication to subtype canine epilepsy or to identify early comorbidities such as epilepsy plus cognitive dysfunction syndrome.

#### 3.6.3. Methodological and Computational Integration

The essential contribution linking the two previous levels and projecting them into a common operational framework belongs to Levitt and the collaborators [45]. The authors developed a unified pipeline of preprocessing, feature extraction and classification applicable to both human and canine and murine EEG. Using the same Support Vector Machine (SVM) algorithm and a small set of statistical features, they achieved comparable performance in separating clean from artifact-laden EEG across species. This feasibility demonstration suggests that, at least for the artifact-detection task studied, species-specific differences may not require fundamentally different algorithmic approaches, but only minimal hardware standardization [44] and comparable registration paradigms. In addition, the existence of developmental normative data for the dog [68] and consolidated human databases [3] creates the prerequisites for using standardized z-scores as a common language between species.

#### 3.6.4. Interim Synthesis and Implications for Translational Research

The three levels of correspondence described above are consolidated, together with the corresponding gaps, in the cross-species synthesis presented in Section 9. Taken together, they place the dog as an integrated component of translational epilepsy research rather than as an analog model, and they identify the single element still missing: coherence, the biomarker on which the argument of this review rests, has not yet been characterized in canine epilepsy itself.

## 4. Acute Brain Injury and Neurocritical Care

Unlike epilepsy, the application of qEEG in acute brain injury—traumatic brain injury, subarachnoid hemorrhage, and hypoxic–ischemic encephalopathy—remains virtually unexplored in veterinary medicine. This section analyzes the asymmetry between the human field, where there are both well-developed theoretical frameworks and validated clinical applications, and the canine one, where the studies are completely lacking. Starting from the fundamental principles established in the human literature—anchoring in normative, separation of pharmacological effects, and validation of cross-species biomarkers—we will argue that this gap is, paradoxically, a major opportunity for translational research. The dog, already validated as a model in epilepsy and benefiting from a neuroanatomy comparable to the human, can become an essential bridge between rodent models and the human patient in neurocritical care.

### 4.1. The Current Status of Canine Research in Acute Brain Injury

#### Absence of Canine qEEG Studies in Acute Pathology

The analyzed database does not include qEEG studies explicitly dedicated to acute brain lesions in dogs—whether it is traumatic brain injury (TBI), subarachnoid hemorrhage, hypoxic–ischemic encephalopathy or status epilepticus. This absence is not surprising, but rather reflects the current state of the field: unlike canine epilepsy, where qEEG is already well characterized and validated as a functional biomarker, veterinary neurocritical care remains insufficiently standardized electrophysiologically.

However, the absence of evidence does not mean the absence of relevance. The dog also has considerable translational potential in this area, for a simple but fundamental reason: the principles of qEEG analysis, quantitative biomarkers and methodological frameworks are already rigorously defined in the human studies included in the analysis. What is lacking is not the theoretical framework, but its systematic application to the canine species in acute contexts.

### 4.2. Translational Foundation of Human Studies

#### EEG Norms and Physiological Landmarks

Any quantitative assessment of the pathological brain assumes the existence of a normal landmark. Terry et al. [66] provided essential data on EEG patterns at various stages of sleep and wakefulness in healthy subjects, thus providing indispensable physiological milestones for the interpretation of changes in acute encephalopathy. These milestones are all the more important as acute brain injury profoundly disrupts the architecture of sleep, and differentiation between pathological patterns and extreme physiological variants requires precise mapping of the normal. Complementarily, Thatcher [3] developed and validated methods for assessing the reproducibility of qEEG and the construction of normative databases. Its key contribution is to demonstrate that pathological deviations can be expressed quantitatively as standardized scores relative to the normal, thus allowing objective and longitudinal monitoring of critically ill patients. This approach—using qEEG scores to quantify severity and guide clinical decisions—is exactly the framework currently lacking in emergency veterinary medicine.

### 4.3. Pharmacodynamic qEEG in the Context of Intensive Therapy

One of the major challenges of EEG monitoring in intensive therapy is the almost-constant presence of neuroactive medication. Patients with acute brain injury frequently receive antiepileptics, sedatives, painkillers and sometimes neuromuscular blockers—all with the potential to profoundly alter EEG patterns.

Clemens et al. [53] demonstrated that different antiepileptics (carbamazepine, valproic acid, and so on) induce specific and reproducible qEEG changes, affecting both spectral power and network dynamics. These “pharmacological fingerprints” are essential in neurocritical care, where the patient receives multiple classes of drugs at the same time, and qEEG must differentiate between the pharmacological effects and those of the brain injury.

In the same direction, Arzy et al. [18] explored dynamic changes in brain networks induced by pharmacological and electrical interventions, helping to understand how brain connectivity changes under the influence of therapeutic agents. This dynamic perspective is crucial for the correct interpretation of EEG in the context of clinical and therapeutic fluctuations specific to intensive therapy. Continuous quantitative monitoring can also capture abrupt, life-threatening events: Mullaguri et al. [69] reported that qEEG neurotelemetry detected impending brainstem herniation before overt clinical signs, illustrating the value of real-time quantitative trends in the neurocritical setting—a capability that could be transposed to the monitoring of critically ill canine patients.

### 4.4. Sleep, Comorbidities and Encephalopathy

The critical patient is not only a damaged brain, but also an organism with a medical and psychological history. Steiger & Kimura [70] pointed out that affective and sleep disorders produce specific qEEG signatures, which may persist or exacerbate in the context of acute injury. This observation has major practical implications: the interpretation of post-injury EEG should consider possible pre-existing comorbidities, which can significantly influence electrophysiological patterns and mimic or mask the signs of acute encephalopathy.

### 4.5. Translational EEG Biomarkers: Cross-Species Validation

The central piece of translational argumentation is provided by Sidorov et al. [71], who validated an EEG biomarker model applicable to both humans and animals. Their study demonstrates the consistency of delta activity between species under comparable pathological conditions, thus providing a directly applicable methodological framework in acute brain injury. Delta diffuse activity—a classic marker of encephalopathy—thus becomes the ideal candidate for a translational biomarker of severity and prognosis. The translational credentials of delta-band measures are further strengthened by work on the Angelman syndrome, a monogenic disorder with well-characterized delta abnormalities. Building on the parallel mouse–human analysis of Sidorov et al. [71], quantitative studies have shown that delta power correlates with cognitive function and with the severity of motor, communication and epilepsy phenotypes [72,73], and that it is reliably measurable even in short recordings and sensitive enough to track antisense-oligonucleotide treatment effects longitudinally [74]. These properties—robustness, clinical correlation and treatment sensitivity—are precisely those expected of a cross-species biomarker, and provide a template against which canine delta-based markers can be evaluated.

This cross-species validation is all the more valuable as it paves the way for preclinical studies in dogs using the same quantitative metrics as human clinical trials, allowing for direct comparability of results.

### 4.6. Canine Relevance in the Context of Emerging Animal-Model Evidence

Translational research into acute brain injury and post-traumatic epilepsy has seen significant expansion in recent years, but it remains focused predominantly on rodent models. Although rat or mouse models are valuable for elucidating molecular and cellular mechanisms, the extrapolation of results in humans is limited by major neuroanatomical differences—especially in terms of encephalization, the ratio of white to gray matter, and the complexity of functional networks. This limitation has led to a growing recognition of the need to integrate species with higher encephalization, such as the dog, which can serve as complementary links between simple rodent models and human clinical complexity.

A strong argument in favor of extending these investigations to the dog comes from Komoltsev et al. [75] who conducted a translational study that included both human patients with acute traumatic brain injury, and a rat model with fluid-percussion injury. The researchers found a remarkably high incidence of epileptiform activity in the acute post-traumatism period in both species. More importantly, in rats, the incidence of epileptiform spikes correlated significantly with microglia density and neuronal loss in the ipsilateral hippocampus. This correlation suggests a direct link between acute electrical activity and secondary structural injury—which means that early epileptiform discharges are not a mere epiphenomenon, but an active pathogen contributing to neuronal degeneration through inflammatory and excitotoxic mechanisms. Komoltsev’s data therefore plead not only for the relevance of early EEG monitoring, but also for the need to extend these investigations to species with cortical organization closer to that of the man, such as the dog.

Another essential dimension, with direct implications for the development of neuroprotective interventions, is represented by the therapeutic window—the interval between trauma and the onset of epileptic seizures. Ronne-Engström et al. [76] continuously monitored through the EEG a group of 70 patients with traumatic brain injury and reported a seizure incidence of 33% with an average interval of 74 ± 47 h post-traumatism. This therapeutic window is of crucial importance as it provides a quantifiable timeframe in which the administration of prophylactic agents could prevent seizures and thereby limit secondary injury. Animal models allow detailed investigation of this window, and the dog—by similarity with the man in terms of post-traumatic dynamics and by the possibility of EEG registration on the scalp for long periods—is a privileged model for testing such therapeutic strategies.

However, the translation of results from animal models to human clinical practice is not without methodological challenges. A careful analysis of these difficulties is provided by Pyrzowski et al. [77], who, in their systematic review of EEG biomarkers for the prediction of post-traumatic epilepsy, emphasize both recent progress and persistent obstacles. These include short observational periods in animal studies, which do not capture the entire course of the disease; difficulties in translating findings from intracranial records in animals to the human scalp EEG, whose spatial resolution is much lower; and the need to integrate quantitative analysis of background activity alongside the assessment of epileptiform activity and sleep abnormalities. These challenges point to exactly the directions in which the canine model can bring added value: the dog allows EEG recordings on the scalp, directly comparable to human ones, over long periods of time, thus reducing the methodological gap between rodent models and the human clinic.

Finally, an essential contribution from the perspective of pediatrics, with direct relevance to the canine model, comes from Benedetti et al. [78]. The study showed that in children in critical condition, exposure to sedatives and analgesics is associated with increased use of continuous EEG and reduced incidence of electrographic seizures. This finding is highly relevant for translational research on the dog, since in this species sedation is almost always required for EEG records either for animal welfare reasons or for the reduction of motion artifacts. Benedetti’s data thus provide a reference framework for interpreting the effects of sedation on quantitative parameters and the sensitivity of detecting epileptiform activity, warning that the absence of electrographic seizures in a sedated animal does not exclude their presence in the waking state. Overall, this emerging evidence places the canine model in a strategic position in the architecture of translational research not as a substitute for rodents, but as a necessary bridge between the basic mechanisms elucidated in the simple models and the clinical complexity of the human patient.

### 4.7. Translational Human–Dog Model in Acute Brain Injury

Based on the studies analyzed, a translational qEEG model is outlined in acute brain injury, built on three fundamental principles.

The first principle is anchoring in normative data. The use of qEEG databases and connectivity metrics in healthy populations allows for objective definition of pathological deviations. For the dog, this involves building normative databases specific to breed, age and registration protocol, similar to those existing for humans [3,56,66]. Without these milestones, the interpretation of changes induced by acute injury inevitably remains subjective and difficult to compare between studies.

The second principle is aimed at separating the pharmacological effects from those of the lesion. Intensive care patients—human or canine—invariably receive neuroactive medication that alters EEG patterns. The studies of Clemens [53] and Arzy [18] provide a framework for identifying pharmaco-specific “fingerprints”, thus allowing for the deconvolution of the EEG signal and the isolation of components related to the brain injury itself. The application of this framework to the dog requires systematic pharmaco-EEG studies, similar to those existing in humans.

The third principle, which is most important from a translational perspective, is the validation of biomarkers in parallel in humans and animals. The paradigm proposed by Sidorov et al. [71]—demonstration of biomarker consistency (e.g., delta activity) between species—should be extended to the entire set of qEEG metrics relevant to acute injury: spectral power in slow bands, cross-frequency coupling, interhemispheric coherence, and stimulus reactivity.

### 4.8. Future Directions

The synthesis of the literature reveals a significant asymmetry between the human and canine fields in terms of the application of qEEG in acute brain injury. While in humans there are both well-developed theoretical frameworks [3,56,66], as well as validated clinical applications [54,55,76,77], in the dog the field remains practically unexplored.

This gap is, paradoxically, a major opportunity. The dog—already validated as a natural model in epilepsy [35,36,79] and benefiting from a neuroanatomy comparable to the human—can become an essential bridge between rodent models and the human patient in the neurocritical setting. Unified methodological platforms, such as the one proposed by Levitt et al. [45] for cross-species EEG classification, can be extended to include specific acute injury metrics.

Future research directions should include: (1) the construction of canine qEEG normative databases, stratified by age and breed; (2) systematic pharmaco-EEG studies to characterize the effects of sedatives and common antiepileptics in dogs; and (3) the development of quantitative analysis algorithms adapted to the specificity of the canine EEG, but compatible with the metrics used in human studies.

The absence of canine qEEG studies in acute brain injury thus defines a priority direction of translational research, with the potential to accelerate both the development of prognostic biomarkers and the testing of therapeutic interventions under controlled but clinically relevant conditions.

Beyond the conceptual framework set out above, two technological developments already available in veterinary neurology outline what a canine program in this domain could look like in the short term. The first is continuous rather than intermittent recording. Human neurocritical care has moved from short punctual records to continuous EEG precisely because paroxysmal activity and slow-wave abnormalities evolve over hours to days after the insult, which is also the interval over which post-traumatic seizures appear [76]. The technical obstacle to doing the same in the dog has largely been removed: implantable devices have been used to record continuously from epileptic dogs living in their own homes [80], and the surgical placement of such devices has itself been compared systematically [81]. Applied to acute injury rather than to chronic epilepsy, the same platforms would allow the trajectory of qEEG parameters to be followed throughout the period in which secondary injury develops.

The second is that the clinical material already exists, and that its natural history is quantified. Traumatic brain injury is not rare in dogs, arising commonly from road traffic accidents and bite wounds; in a series of 235 dogs presented with severe blunt trauma, a quarter carried a clinical diagnosis of head trauma, and its management, comprising fluid therapy, hyperosmolar agents, analgesia, anticonvulsants, oxygen supplementation and, where indicated, surgery, corresponds closely to human neurocritical practice [82]. More pertinently for a review concerned with epilepsy, the canine sequelae parallel the human ones: about 7% of dogs sustaining head trauma develop epilepsy within the following year, the risk rising with injury severity and reaching some 15% in animals with skull fractures, against reported human figures of 2% after mild, 4% after moderate and 15% after severe injury, and about 14% of dogs show seizures within the first 24 h, early seizure activity being associated with higher mortality [82]. These are precisely the outcomes that continuous quantitative monitoring would be expected to anticipate, and they are already documented in the dog without any electrophysiological correlate having been sought. The obstacle to applying qEEG in this setting is therefore methodological and technological rather than a shortage of cases, which distinguishes this gap from those in which the canine phenotype itself is uncommon.

Taken together, these elements suggest a concrete sequence: pilot studies applying continuous recording, on platforms already validated in epileptic dogs, to animals hospitalized for head trauma or status epilepticus, timed against the post-traumatic risk window described in human patients; the construction of canine qEEG classifiers, trained and reported separately by lesion type rather than pooled, since the qEEG signature of a dysfunction is unlikely to be independent of its cause; and prospective registries linking qEEG trends to severity scores and clinical outcome, using the z-score logic established for the human domain by Thatcher [3].

A second consideration concerns how such recordings should be analyzed. In a study of 460 video-EEG recordings from 71 patients monitored in intensive care, the best classifier applied to the sample as a whole reached a mean F1 score of 0.76, whereas models selected and trained within etiological subgroups performed appreciably better, reaching 0.84 for cranioencephalic trauma, 0.84 for cerebral hemorrhage and 0.89 for cardiorespiratory arrest [83]. The authors concluded that performance varies with the cause of the seizure and that tailored approaches are therefore required. The principle bears directly on the canine case, and it qualifies the cross-species portability demonstrated in Section 3: an algorithm may transfer between species while still requiring stratification by lesion type within each of them, so canine classifiers for acute injury should be trained and reported by etiology rather than pooled.

## 5. Neurodegeneration, Cognition and Aging

Aging of the population, both human and canine, has brought to the fore the need for objective tools for assessing brain health throughout life. This section follows the continuum of development and cognitive decline, integrating data on the maturation of EEG in young dogs with those on changes associated with normal and pathological aging in humans. We will explore how canine cognitive dysfunction syndrome (CCD) is validated as a spontaneous model of dementia, with qEEG and sleep patterns remarkably similar to those described in Alzheimer’s disease and in the early stages of human cognitive decline. From developmental norms to translational biomarkers of neurodegeneration, this section outlines a common map of brain aging in the two species.

### 5.1. Canine Component—Development, Aging and Cognitive Dysfunction

#### 5.1.1. EEG Maturation Throughout Life

Pellegrino and Gómez Álvarez [68] conducted a prospective observational study in healthy dogs of different ages, with the aim of characterizing the maturation of background EEG and spectral composition during development. The research highlighted systematic changes in the dominant frequency and spectral power distribution by age, demonstrating that qEEG parameters follow a predictable evolutionary trajectory. Significant correlations were also identified between EEG patterns and the neurobehavioral developmental stages of dogs. By providing the first normative data for the canine species, this study provides an essential tool for discriminating between normal physiological maturation and pathological deviations, such as those encountered in encephalopathies or canine cognitive dysfunction syndrome. The reliability of the sleep measures underpinning later aging studies has itself been examined: Gergely et al. [84] showed that sleep-stage scoring in family dogs was reproducible using both manual and automated identification, providing a methodological foundation for quantitative sleep-EEG studies of canine aging and cognition.

#### 5.1.2. Canine Cognitive Dysfunction (CCD)—A Natural Model of Dementia

qEEG research in the field of canine cognitive dysfunction is fundamentally based on two consecutive investigations conducted by Mondino’s group. These studies have transformed the CCD from a descriptive clinical entity into a well-characterized electrophysiological biomarker model relevant to both veterinary medicine and translational research on human dementia.

In the first study, published in 2022, Mondino et al. [85] adopted a case–control design to compare three categories of dogs: 12 normal dogs, seven dogs in a stage of “at risk” (with increased risk for cognitive decline but still without established diagnosis) and six dogs with clinically confirmed canine cognitive dysfunction syndrome. EEG recordings were made in a state of wakefulness, using subcutaneous needle-type electrodes—a method that minimizes discomfort and movement artifacts. The results revealed distinct electrophysiological patterns for each group. In dogs “at risk”, an increase in spectral power in the alpha band at the frontopolar level was observed, a subtle change that could represent an early signal of decline. In dogs with confirmed CCD, instead, a different qEEG pattern was outlined, characterized by localized changes in the frontal and temporal regions. To validate the functional significance of these findings, the authors conducted a correlational analysis, demonstrating a significant association between qEEG parameters and cognitive test scores. This correlation confirms that electrophysiological changes are not artifacts without biological significance, but directly reflect the degree of cognitive impairment.

Extending this line of research, Mondino et al. [86] investigated an observational cohort of elderly dogs, moving the focus from wakefulness to sleep architecture. The study looked at how polysomnographic parameters and sleep qEEG correlate with cognitive impairment. The results were conclusive in three major directions. First, there was a significant reduction in NREM and REM sleep duration in dogs with more severe cognitive impairment, a well-known phenomenon also in human dementia. Second, spectral sleep analysis revealed consistent alterations in spectral power, particularly in delta and theta bands (frequency bands traditionally associated with sleep depth and mnesic consolidation processes). Thirdly, the study identified significant correlations between these electrophysiological changes and performance on cognitive tests, thereby strengthening the environmental validity of the measurements.

Overall, the two studies of the Mondino group [85,86] transform polysomnographic parameters and sleep qEEG from simple physiological descriptors into functional biomarkers of cognitive decline and brain aging in dogs. They also open up the prospect of using the dog as a natural model for human forms of dementia (especially Alzheimer’s disease) in which sleep alterations and EEG spectral changes are considered early markers and correlated with disease progression.

### 5.2. Human Component—Aging, Dementia and Cognitive Impairment

#### 5.2.1. Normal Aging and Functional Connectivity

A thorough understanding of the brain changes associated with physiological aging is essential to distinguish between normal and pathological cognitive decline. In this context, Terry, Anderson and Horne [66] conducted a study on healthy human subjects, focusing on NREM sleep and EEG functional connectivity. Research has shown that physiological aging is accompanied by a decrease in functional connectivity and a reorganization of brain networks during sleep, which is likely to reflect a loss of effective integration between cortical regions. More importantly, these electrophysiological changes correlate both with the subjective quality of sleep reported by participants and with age-dependent cognitive measures, thereby strengthening the functional validity of connectivity parameters. By providing a quantitative age-based EEG connectivity benchmark, the study provides an essential benchmark for translational research: without such regulatory data, any attempt to quantify pathological deviations in dementia or other cognitive disorders risks confusing the effects of normal aging with specific disease signatures. For the canine model, this methodological framework is directly applicable, allowing the development of similar landmarks in the dog and paving the way for comparative studies of brain connectivity in normal and pathological aging.

#### 5.2.2. Alzheimer’s Disease and the Spectrum of Cognitive Decline

The validation of qEEG as a diagnostic tool in Alzheimer’s disease requires not only the identification of specific biomarkers, but also the demonstration of their ability to discriminate between different stages of the disease and reflect the degree of cognitive impairment. Three major studies offer, from this perspective, convergent and complementary contributions.

Garn et al. [59] investigated patients with early to moderate Alzheimer’s disease, looking at qEEG based on cognitive status rest, cognitive load and other experimental conditions. Identified qEEG biomarkers include an increase in spectral power in slow bands (delta and theta), a reduction in alpha band power, as well as changes in functional connectivity. A key finding of the study is that these markers, although dependent on the cognitive state of the patient, are reproducible and, more importantly, correlate significantly with the severity of cognitive impairment. This correlation validates qEEG—not only as a transversal diagnostic tool but also as a potential marker of longitudinal progression.

Extending this comparative approach, Wang et al. [22] conducted a case-type study of control, comparing patients with Alzheimer’s disease diagnosed with healthy controls. The research described a set of qEEG biomarkers including spectral power in different bands and frequency ratios that discriminate with significant accuracy between the two groups. Based on these results, the authors propose qEEG as a complementary diagnostic tool, useful especially in situations where clinical or imaging evaluation is inconclusive, and as a potential marker of disease progression, capable of detecting subtle functional changes in short intervals.

The most comprehensive contribution of the three, however, belongs to Ferreira et al. [87], whose study, published in Dementia and Geriatric Cognitive Disorders, evaluated a complete cognitive continuum. The group included healthy subjects, patients with subjective cognitive decline (SCD—where the patient reports cognitive difficulties but objective tests are still normal), patients with mild cognitive decline (MCI—intermediate stage between normal aging and dementia) and patients with established dementia. The results showed that qEEG highlights gradual changes across this spectrum, without sudden jumps between categories. This property is of paramount importance: it transforms qEEG into a sensitive marker of early transitions in cognitive decline, capable of detecting functional alterations since the phase of subjective cognitive decline long before the dementia itself became clinically manifest.

Overall, the three studies converge toward the same conclusion: qEEG offers a set of quantitative parameters (spectral power in slow and alpha bands, frequency ratios, connectivity measures) that not only differentiate Alzheimer’s patients from healthy subjects, but are sensitive to the full spectrum of cognitive decline from preclinical stages to advanced dementia. For the canine model, these findings are directly relevant because canine cognitive dysfunction syndrome (CCD) exhibits neuropathological and behavioral similarities with human Alzheimer’s disease. Validation of the same qEEG biomarkers in dogs would allow not only early diagnosis and monitoring of CCD progression, but also testing of therapeutic interventions within a natural dementia model with direct translational potential to human medicine. The applicability of qEEG within dementias is not limited to Alzheimer’s disease. Stylianou et al. [88] showed that EEG slowing and dominant-rhythm variability distinguished dementia with Lewy bodies and Parkinson’s disease dementia from Alzheimer’s disease and from controls, and that these measures tracked cognitive fluctuations, classifying Lewy body from Alzheimer dementia with high sensitivity and specificity. Because canine cognitive dysfunction encompasses heterogeneous neuropathologies, this capacity of qEEG to separate dementia subtypes may prove relevant for phenotyping the canine syndrome.

#### 5.2.3. Vascular and Post-Viral Cognitive Impairment

Extending the applicability of qEEG beyond primitive neurodegenerative pathologies, recent research has explored its ability to capture functional signatures of cognitive decline in vascular and post-viral etiology. Two studies conducted in the context of stroke and one in the post-COVID-19 context provide convergent evidence in this regard.

In the field of vascular etiology, Song et al. [89] conducted a longitudinal cohort study on patients who suffered a cerebral infarction, aiming at the further development of cognitive deficit. The research identified two significant qEEG predictors: a background rhythm frequency (background rhythm frequency, BRF) of less than 7.4 Hz and an increase in spectral power in the theta band in the EEG basic. These parameters, recorded in the acute phase post-infarct, accurately predicted the occurrence of cognitive decline during longitudinal tracking, thus transforming qEEG from a simple electrophysiological descriptor into a valid predictor of unfavorable cognitive vascular cause.

Expanding this paradigm, Finnigan et al. [90] used qEEG to stratify the risk of cognitive deficit in the post-stroke context. The study demonstrated the usefulness of quantitative parameters in guiding prognosis and therapeutic decisions, providing physicians with objective tools to identify early patients at high risk of cognitive decline and to adapt rehabilitation and monitoring strategies accordingly. Converging evidence from the same group was provided by Schleiger et al. [91], who showed that post-stroke qEEG informs the early prognostication of cognitive impairment, reinforcing the notion that quantitative slowing measured in the acute phase carries prognostic information, a principle whose application to canine cerebrovascular disease remains to be tested.

A completely new direction, generated by the COVID-19 pandemic, was explored by Gaber et al. [9], who investigated patients with cognitive deficit post-COVID-19 (an increasingly common clinical entity, known as “post”-viral cognitive fog). The study identified a significant increase in spectral power in the theta band in the frontal, central and parietal regions in these patients compared to healthy controls. More importantly, these electrophysiological changes significantly correlated with performance in neuropsychological tests of verbal learning and executive functions, thus strengthening the evidence that post-viral cognitive impairment is not only a subjective phenomenon, but has an objective functional signature, detectable by qEEG.

Overall, these three studies broaden the scope of qEEG’s applicability beyond epilepsy and primary neurodegenerative diseases, demonstrating its sensitivity to cognitive impairment of various etiologies, vascular and post-viral. For the canine model, these findings are relevant to the extent that the dog can develop forms of cognitive impairment secondary to other pathologies (e.g., post-stroke or post-viral infections), and qEEG could provide the same tools for predicting, stratifying risk and monitoring evolution as in human medicine.

#### 5.2.4. Development and Maturation—The “Young” End of the Continuum

The cognitive continuum is not limited to the decline associated with aging and disease; the opposite end (development and maturation of the nervous system) provides an equally important framework for understanding functional changes reflected by qEEG. Two studies on healthy human populations, one in adults and another in infants, complement this perspective and align with recent results in the dog.

Kaplan et al. [60] conducted a large-scale epidemiological study on middle-aged and older adults, aiming to identify the best predictors of subjective sleep quality. The results revealed an initial counterintuitive finding: classic sleep architecture parameters such as the duration and continuity of phases more accurately predict the subjective perception of sleep quality than spectral qEEG markers. This dissociation between objective changes in the EEG signal and subjective experience reported by the participant has important methodological implications beyond human medicine. In particular for the interpretation of behavior in nonsedated animals, where we do not have a subjective verbal report, it is essential to recognize that objective EEG parameters do not always overlap perfectly with perceived internal states, a limit which translational studies must consider.

At the opposite end of the age spectrum, Markovic et al. [92] investigated healthy infants, focusing on NREM sleep during the critical period of early postnatal maturation. Research has shown that at this age, NREM sleep is characterized by an increased frontal delta coherence, an electrophysiological pattern that reflects a still-immature functional organization of EEG networks, different from the mature adult pattern. The study also showed that these connectivity parameters are associated with developmental parameters and environmental factors, suggesting that the functional architecture of the maturing brain is shaped by both endogenous and exogenous factors.

These two investigations together provide an essential physiological reference framework. The sensitivity of sleep qEEG to environmental factors was illustrated by Dunbar et al. [93], who found distinct EEG power-spectral responses to wind-farm versus road-traffic noise during sleep, a reminder that the recording environment itself can shape quantitative sleep parameters and must be controlled in translational comparisons. These two investigations together provide an essential physiological reference framework for the interpretation of the results obtained by Pellegrino and Gomez Alvarez [68] in the dog. In both species, human and dog, there is a common pattern: cognitive and neurobehavioral maturation is accompanied by a deep reorganization of the EEG networks, visible both in the waking state and in sleep. More importantly, deviations from this physiological pattern of development, whether it is delayed maturation in childhood or alterations in the architecture of sleep in the elderly, may signal the presence of neurological pathology or an increased risk of cognitive decline. Thus, studies on the heads of “young” and “old” of the continuum of development converge to the same conclusion: qEEG provides a quantitative window on the functional state of the brain, with sensitivity to deviations both below and above the normal trajectory.

### 5.3. Translational Human–Dog Model in Neurodegeneration, Cognition and Aging

#### 5.3.1. Normative Data and Development

The integration of data from canine and human studies outlines a coherent translational model in which the quantitative parameters of the EEG serve as a common language for describing maturation and brain aging. In the dog, Pellegrino and Gomez Alvarez [68] first described the trajectory of EEG maturation throughout development, providing age-specific normative milestones. In parallel, in humans, Kaplan et al. [60] investigated the correlations between sleep architecture and subjective perception in adults, while Markovic et al. [92] characterized frontal delta coherence patterns in healthy infants, reflecting the immature functional organization of EEG networks. Together, these studies define the physiological trajectory of the development and aging of brain networks in the absence of pathology from infants to older adults, and respectively from puppies to aged (geriatric) dogs. This normative basis is essential for the interpretation of pathological deviations: only through detailed knowledge of age-related physiological changes can it be established whether an alteration in the parameters of qEEG signals the presence of dementia in humans or canine cognitive dysfunction syndrome (CCD) in dogs. Thus, the similarities of transspecies in terms of development and aging trajectory validate the dog as a natural model for the study of neurodegenerative processes and brain aging.

#### 5.3.2. Dementia and Cognitive Decline—Spontaneous Patterns and Spectrum

The parallel extends from normative data to the pathological patterns of cognitive decline. In human patients qEEG detects not only established dementia but the stages preceding it, changing gradually across the continuum from subjective complaint through mild impairment to dementia [22,59,87]. In the dog an almost parallel gradient has been described, from normal animals through an at-risk stage to confirmed canine cognitive dysfunction, with frontopolar alpha power rising early and sleep architecture and slow-band power tracking severity thereafter [85,86]. The two continua are set side by side in the cross-species synthesis presented in Section 9. What the canine series still lacks is longitudinal follow-up of the same animals, without which the at-risk stage cannot be shown to precede rather than merely accompany decline.

Thus, the dog outlines the same cognitive continuum (normal to “at risk” to CCD) that Ferreira and his collaborators described in humans (healthy—SCD—to MCI to dementia). In both species, qEEG captures early changes or example, the increase in frontal alpha power in dogs “at risk” and the spectral and sleep parameters are sensitive to the degree of cognitive impairment. CCD is thus validated as a spontaneous canine model of dementia, and the structure of the EEG/cognitive continuum shows a remarkable analogy between the two species, reinforcing the translational value of the dog for the study of Alzheimer’s disease and other forms of cognitive decline.

## 6. Methodology and Technology Bridge

Behind every clinical application lies a solid methodological infrastructure, without which qEEG data remain difficult to interpret and hardly comparable between studies. This section defines the technological framework allowing the use of qEEG as a clinical and translational tool, integrating both canine technical validation studies on the influence of electrode type, fitting and physiological status on quantitative parameters and human studies that have set standards for acquisition, spectral analysis, connectivity measures and control of confounding factors. The central part of this section is the demonstration that the same qEEG parameters and computational analysis platforms can be applied comparably in both species, provided that common methodological standards are observed.

**Figure 3:**
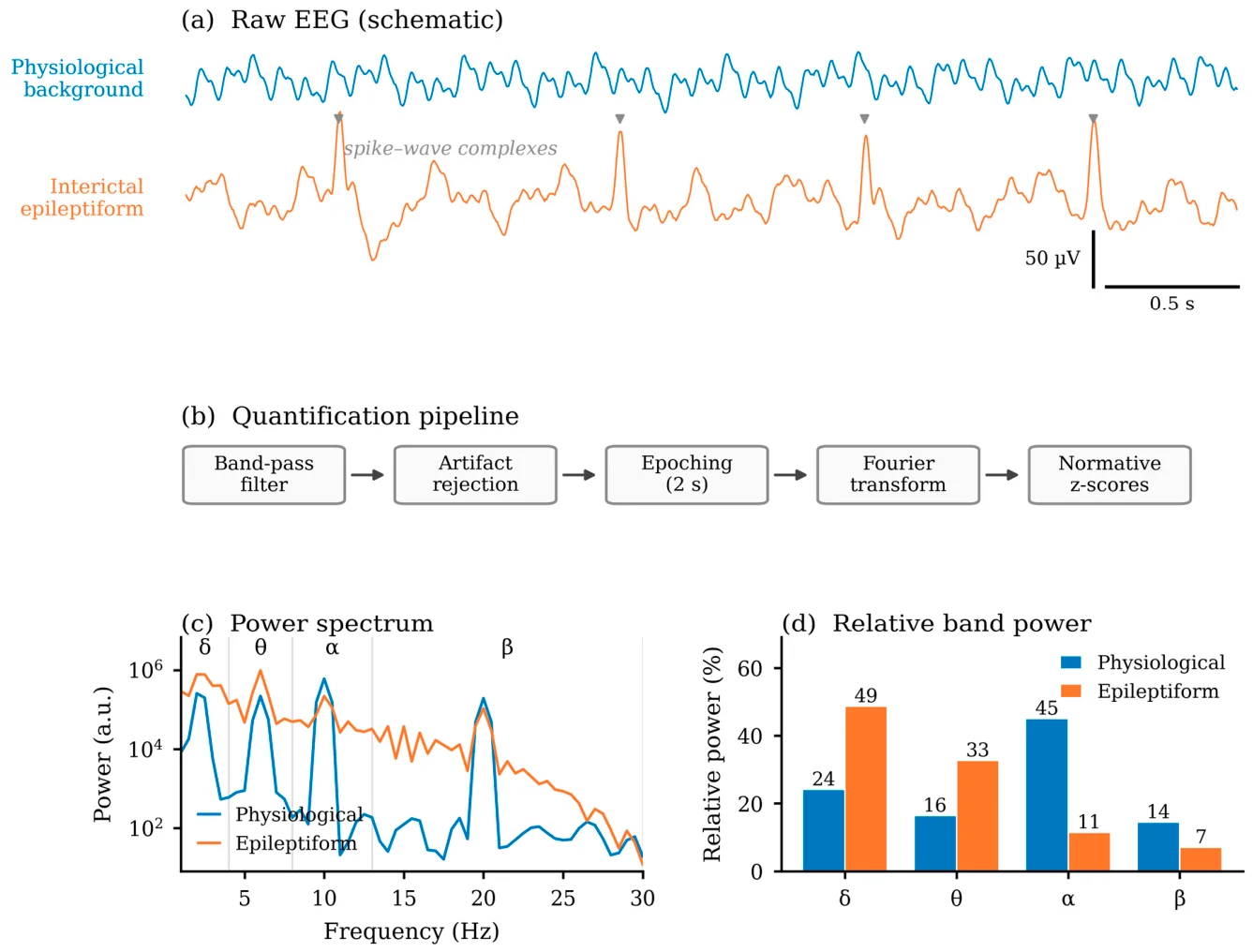
Schematic illustration of the conversion of the raw EEG signal into quantitative descriptors. (**a**) Four seconds of physiological background activity, dominated by an alpha rhythm, and of interictal epileptiform activity in which spike–wave complexes are superimposed on a slowed background. (**b**) The successive processing steps: band-pass filtering, rejection of artifacted segments, division of the record into analysis epochs, Fourier transformation, and expression of the resulting values as z-scores against an age-matched normative database. (**c**) Power spectra of the two traces, with the conventional band limits indicated. (**d**) Relative band power derived from the same spectra, showing the increase in delta and theta and the reduction in alpha that characterize the epileptiform record. The traces are synthetic and are shown to illustrate the analytical sequence; they are not clinical recordings, and the values in panels (**c**,**d**) are computed from the traces in panel (**a**) rather than taken from any published dataset.

Because the remainder of this section concerns the conditions under which these numbers can be trusted, it is useful to recall briefly how they are obtained. The raw signal is band-pass filtered, segments contaminated by movement, muscle or ocular activity are removed, and the remaining record is divided into epochs of fixed length. Each epoch is transformed into the frequency domain, most commonly by a Fourier transform, and the resulting power is summed within the conventional frequency bands to yield absolute and relative power; coherence is computed between pairs of derivations from the same transformed epochs, and the values may finally be expressed as deviations from an age-matched normative database. The sequence is summarized in Figure 3. Two properties of this chain matter for everything that follows: the epoch length fixes the frequency resolution of the analysis, and every step downstream of acquisition inherits whatever was introduced upstream of it, which is why the acquisition conditions discussed below are not a technical preamble but a determinant of the result.

### 6.1. Canine Component: Standardization and Technical Validation

In canine studies, the methodological focus is on the technical feasibility and validity of the qEEG record, as extrapolation of results to humans is critically dependent on rigorous control of procurement conditions. Three major investigations have demonstrated that qEEG parameters in dogs are sensitive to multiple variables: Bocheńska et al. [36] showed that phenobarbital treatment alters the spectral profile in a way dependent on clinical response; Bassett et al. [41] developed standardized models of convulsive provocation with picrotoxin and pentylenetetrazole; and Musteata et al. [44] made an essential methodological contribution by intra-subject comparison of different types of electrodes in canine EEG recordings.

The latter pointed out that the use of frequent needle-type electrodes in the canine EEG (minimally invasive character and the ability to reduce motion artifacts) significantly influences the measures of coherence and functional connectivity. This finding imposes particular caution in interpreting network analyses in dogs and stresses the need for rigorous hardware standardization in all translational studies aimed at direct comparison between species. More recently, Hermándy-Berencz et al. [21] obtained interpretable non-invasive recordings in epileptic dogs without sedation, confirming that canine EEG can be acquired under conditions closer to those of human practice and thereby narrowing one of the principal methodological gaps between the two species.

Beyond the technical aspects of electrodes and assembly, pharmacological effects on EEGs are another major source of confusion, relevant to both human and veterinary medicine. Although derived mainly from studies on human subjects, Arzy et al. [18], Steiger and Kimura [70] and Garn et al. [59] demonstrated that antiepileptic drugs and psychoactive agents produce consistent and reproducible qEEG changes: increased power in certain bands, reduction in others, and changes in coherence that can either mimic pathological signatures or mask them completely.

These results have direct implications for veterinary medicine, where canine patients are frequently subjected to chronic antiepileptic treatment regimens. A phenobarbital-therapy dog, for example, may have a profoundly pharmacologically modified EEG pattern with spectral redistributions similar to those described by Bochenska that should not be confused with an aggravation of the underlying pathology or an independent change in the condition of the disease. Thus, both hardware standardization (electrode type, assembly) and strict control of pharmacological status at the time of registration are sine qua non conditions for the validity and comparability of qEEG data in human–dog translational research. The scope of current canine practice was mapped by Luca et al. [94], whose survey of EEG usage and techniques in dogs documented the persisting heterogeneity of electrodes, montages and sedation protocols across centers, underscoring the standardization gap that must be closed before canine qEEG data can be pooled or compared across laboratories. It is worth recalling that quantitative EEG was applied to the dog remarkably early: Jones and Greufe [95] described a quantitative electroencephalographic method for xenobiotic screening in the canine model, anticipating by two decades the pharmaco-qEEG approaches later pursued in canine epilepsy.

### 6.2. Human Component—Standards, Normative Data and Advanced Analysis

In contrast to the still-pending standardization efforts in the veterinary field, the qEEG methodology in human studies is considerably better defined, thus providing a robust benchmark for the development of the canine equivalent. This methodological maturity manifests itself on multiple levels: from theoretical frameworks that guide the interpretation of connectivity measures, to normative databases that allow objective quantification of pathological deviations, and to advanced techniques of computational analysis and automatic classification. Acquisition technology is evolving in parallel: Fiedler et al. [96] validated a novel multipin dry-electrode cap for electroencephalography, a development that reduces set-up burden and could, in principle, ease the practical constraints of recording in less cooperative subjects—an appealing prospect for veterinary application.

A key theoretical basis for the correct use of qEEG was developed by Srinivasan et al. [57], who developed a rigorous framework for the interpretation of the EEG spectrum and coherence measures. Their work demonstrates how neural sources and volumetric conduction phenomena, that is, how the electrical currents generated in the brain propagate through the tissues up to the scalp, can profoundly influence the measures of functional connectivity. This contribution is fundamental to avoiding one of the most common methodological pitfalls in both clinical and translational studies: over-interpretation of EEG coherence as direct evidence of communication between distinct brain regions. Understanding that two EEG signals may seem coherent not because the respective “regions communicate actually”, but because they share the same neural source or are affected by the same conduction artifact, is essential for any researcher using these metrics either in humans or in dogs.

Beyond the theoretical framework, the clinical applicability of the standardized qEEG was underlined by Walker [58], especially in the context of neurofeedback therapy in patients with epilepsy and comorbid disorders. The author pointed out that the effectiveness of therapeutic interventions directly depends on the accuracy of measurements and compliance with analytical standards, a conclusion with universal validity: without a solid methodological basis, even the most sophisticated intervention cannot be properly evaluated, and the results obtained under non-standard conditions risk being irreproducible.

A central pillar of the qEEG methodology in human medicine is the existence of solid norms and reproducibility data. Works coordinated by Thatcher [3] demonstrated that qEEG parameters are reproducible both intra-session and between separate registration sessions, and can be reported to normative databases using standardized scores (z-score). This framework allows for the objective interpretation of functional deviations (i.e., a delta power increased by 2.5 standard deviations from the average population of the same age) and constitutes a common language between studies, between laboratories and, crucial for translational research, between species. A z-score of +2.5 for delta power in a particular region means the same: a significant deviation from the norm regardless of whether it is calculated for a human or a dog, provided that there are appropriate norms for each species (such as those provided by Pellegrino and Gomez Alvarez, 2023, for the dog) [68]. The technical preconditions for such comparability have since been codified: the International QEEG Certification Board guideline specifies minimum requirements for acquisition, visual inspection, epoch selection, artifact rejection and metric computation, and makes explicit that the validity of any qEEG metric depends on the quality of the underlying recording [14].

In parallel, Clemens et al. [53], Arzy et al. [18] and Garn et al. [59] defined the concept of pharmacodynamic qEEG, showing that different classes of drugs as antiepileptics, antidepressants, and anxiolytics induce “specific EEG prints, characterized by changes in spectral power, dominant frequency and coherence”.

These results have direct methodological implications, demonstrating that the interpretation of qEEG without rigorous control of concomitant medication may lead to erroneous conclusions, a warning as valid for the human clinician as it is for the veterinarian who monitors a dog receiving phenobarbital or other antiepileptic drugs.

Finally, recent studies of advanced analysis and computational integration such as those of Kaplan et al. [60], Youh et al. [61] and Naim-Feil et al. [62] extend the classic qEEG beyond spectral analysis and coherence, to network metrics (grade, clustering, minimum paths), time dynamics (variability of connectivity over time) and automatic classification using machine learning algorithms. These tools are essential for the functional modeling of the brain under complex clinical conditions, from epilepsy to schizophrenia and from Alzheimer’s disease to mood disorders, and for the development of computer-aided diagnostic algorithms that can reduce the subjectivity of interpretation and increase the reproducibility of diagnosis. For translational research, these advanced techniques, once validated on human normative databases, can be applied directly to canine data, thus accelerating the integration of the dog into the circuit of computational models of neurological and psychiatric diseases.

### 6.3. Translational Human–Dog Model—Methodological Bridge

The construction of a genuine methodological bridge between human and canine research in the field of qEEG is based on a fundamental premise: the same quantitative parameters can be used comparably in both species, but only under the condition of rigorous compliance with common methodological standards. This premise, although simple in the statement, involves a coordinated effort to harmonize protocols, control confounding variables and unify analysis platforms.

The human literature provides some essential landmarks for the construction of this bridge. First, researchers have identified which parameters are truly reproducible and robust. Srinivasan et al. [57] and Thatcher [3] provided a list of metrics (spectral power in frequency bands, consistency, phase), measures that demonstrated stability both intra-session and between separate recording sessions. This reproducibility is a necessary condition for any serious biomarker, since a parameter that varies chaotically from one record to another can serve neither diagnosis nor therapeutic monitoring. Secondly, Thatcher [3] proposed a unitary framework for reporting functional deviations: using z-scores relative to age-adjusted regulatory databases. This system allows a deviation of +2.5 standard deviations for delta power in a particular region to mean the same regardless of the laboratory or species in which it was calculated—provided, of course, that there are appropriate norms for each species. Third, numerous studies including those of Clemens et al. [53], Arzy et al. [18] and Garn et al. [59] showed how major confounding factors can be controlled, in particular the pharmacological effects of antiepileptic and psychoactive medication, as well as age or wake/sleep variability (documented by Terry and collaborators, 2004, and Kaplan and collaborators, 2016 [60,66]).

In dogs, existing methodological studies offer the first pieces of this puzzle, although they have not yet been integrated into a unitary framework comparable to that of human medicine. Musteata et al. [44] demonstrated that the electrode type, in particular the frequent use of needles electrodes, significantly influences the coherence and connectivity measures, imposing a rigorous standardization of the hardware. Bocheńska et al. [36] showed that phenobarbital treatment profoundly alters the spectral profile of canine EEG, providing a clear example of a pharmacological effect to be controlled. Itamoto et al. [30] quantified the dose-dependent effects of sedation with medetomidine, stressing that consciousness alters the EEG spectrum in a way that can mimic or mask pathological signatures. What is currently lacking is the integration of these fragmentary findings into a unitary guide to good practice for canine qEEG, similar to that which exists in human medicine.

A proof of concept that such integration is not only desirable, but also feasible, was provided by Levitt et al. [45]. The authors used the same Support Vector Machine (SVM) algorithm and the same set of statistical features to classify EEG states in humans and dogs, achieving similar performance in both species. This result demonstrates that differences between species do not require fundamentally different algorithmic approaches, but only a careful standardization of signal acquisition and preprocessing. In other words, once the data are brought to a methodologically common denominator, the same algorithms can operate with comparable accuracy on both species.

Overall, the human–dog methodological bridge rests on three main pillars. The first pillar is hardware standardization: choosing types of electrodes and assemblies that minimize the variability introduced by the equipment, as recommended by Musteata et al. [44]. The second pillar is the rigorous control of physiological and pharmacological factors: detailed documentation of age, state of consciousness (wake versus sedation versus sleep) and concomitant medication, essential to avoid confusion between the effects of pathology and the effects of confounders [30,36,53]. The third pillar is the use of common metrics and unified analysis platforms, as proposed by Levitt et al. [45], allowing direct comparability of results between species. These three elements, standardized hardware, confounding control and common metrics, constitute the fundamental methodological infrastructure on which both rigorous veterinary clinical trials and truly comparative translational investigations will be built in the future. Without this infrastructure, the data obtained in the dog remain isolated anecdotes, which are difficult to integrate into the body of knowledge accumulated in human medicine; with it, the dog becomes a genuine partner in translational research of epilepsy and other neurological disorders.

These three pillars can be stated as an explicit reporting standard. Table 1 sets out a minimum specification for the acquisition and reporting of canine qEEG, obtained by adapting the human clinical requirements, as codified by the International QEEG Certification Board and by the joint IFCN–ILAE working group, to the constraints that the canine literature has itself identified. It is offered not as a consensus document, which would require a formal task-force process, but as a starting point against which future canine studies could be designed and appraised, and as the common denominator that any pooling of data across centers would presuppose.

## 7. Neurological Rehabilitation and Neuroplasticity

If until now we have mainly discussed diagnosis and monitoring, this section addresses the dynamic dimension of brain function: the ability of the brain to reorganize itself in response to therapeutic interventions, cognitive training or recovery processes. qEEG occupies a strategic role in this field, because, unlike structural biomarkers, it is sensitive to the dynamic reorganization of neural circuits. We will look at both canine studies documenting functional changes associated with disease progression and response to treatment, and human studies using qEEG to track the effects of neurofeedback, pharmacological therapy, and physical therapy on cortical networks. The convergence of these data suggests that functional plasticity mechanisms are preserved between species, and qEEG can serve as a common biomarker of therapeutic response and recovery.

### 7.1. Canine Component—Functional Plasticity and Adaptation

Although none of the studies analyzed had as a main objective the explicit investigation of neuroplasticity, several longitudinal or comparative observations provide indirect clues on the ability of the canine brain to reorganize its functional patterns in the context of disease progression or therapeutic intervention.

Bocheńska et al. [36] studied dogs with idiopathic epilepsy under phenobarbital treatment in a pre–post longitudinal design. The qEEG analysis showed a significant change in the spectral profile characterized by a decrease in delta band power and a redistribution of the balance between slow and fast bands linked to clinical seizure control. Although the authors do not explicitly discuss neuroplastic mechanisms, these spectral changes support the hypothesis of a functional reorganization of cortical networks induced by treatment, which could overcome a simple acute pharmacological effect. Basically, under the influence of medication, the brain seems to achieve a new state of functional balance, different from both the initial pathological and normal physiological state.

In the context of cognitive decline, Mondino et al. [85] compared normal dogs, “at-risk” dogs (with increased risk for cognitive decline) and dogs with confirmed canine cognitive dysfunction syndrome (CCD). qEEG showed an increase in spectral power in the alpha band at the frontal level in “at-risk” dogs and distinct electrophysiological patterns in those with CCD, significantly correlated with cognitive test scores. The authors suggest that the patterns observed in dogs at risk, especially the increase in frontal alpha power, could reflect early compensatory mechanisms, possibly prior to significant structural changes. These findings indicate that there are functional network changes during the transition from normal to dementia, which could be targets for early therapeutic interventions.

Extending this line of research, Mondino et al. [86] analyzed sleep architecture and qEEG parameters in elderly dogs with varying degrees of cognitive impairment. Reductions in spectral power in the slow-wave band during sleep and altered interhemispheric coherence were associated with severity of cognitive decline. Although the cross-sectional design of the study does not allow one to track the dynamics of changes in the same individual, the significant correlations between CCD severity and EEG parameters support the potential of these metrics to serve as longitudinal biomarkers in future studies. Thus, they are a necessary basis to be able to track, in further research, any functional changes associated with recovery or response to therapy.

### 7.2. Human Component—qEEG as a Marker of Neuroplasticity and Rehabilitation

#### 7.2.1. Neurofeedback, Pharmacological Treatment and Network Reconfiguration

The ability of qEEG to capture functional changes induced by therapeutic interventions, either pharmacological or behavioral, is essential for understanding neuroplasticity mechanisms and for personalized treatment guidance. Three human population studies provide convergent evidence of this, each illustrating a different way of reconfiguring cortical networks.

Fogelson et al. [65] investigated patients with dementia associated with Parkinson’s disease, who received rivastigmine, a cholinesterase inhibitor commonly used in Alzheimer’s disease. The study, a longitudinal open-label study, tracked qEEG changes over a 12-week treatment period. The results showed at the end of this period a significant increase in spectral power in the alpha band and a reduction in activity in the slow bands (delta and theta), changes that significantly correlated with clinically assessed cognitive improvement. Although the authors do not explicitly use the term neuroplasticity, these findings indicate that cholinergic therapy induces a measurable change in electrophysiological patterns, a functional reconfiguration of cortical networks that can be objectively tracked through qEEG.

Another way of inducing network changes in a behavioral nature is illustrated by Walker [58]. The study looked at epilepsy patients undergoing a qEEG-guided neurofeedback protocol, a technique whereby patients learn to and voluntarily modify their own EEG patterns, while receiving real-time feedback. The results demonstrated both a reduction in epileptic seizure frequency and clinical improvements associated with normalization of individual qEEG deviations identified prior to intervention. In this context, qEEG plays a triple role: diagnosis (identification of therapeutic targets), intervention guide (real-time feedback) and response monitoring (post-intervention evaluation). The persistence of EEG changes after the protocol conclusion suggests a sustainable reconfiguration of cortical networks, achieved through operant learning, a classic example of behaviorally induced functional neuroplasticity.

Extending this paradigm to the psychiatric field, Steiger and Kimura [70] conducted a comprehensive review of the literature on EEG of wakefulness and sleep in depression. The synthesis highlights that EEG patterns in particular slow-wave sleep architecture and spectral power in different, bands are systematically modified under antidepressant treatment, for example by normalizing the duration and depth of slow-wave sleep. The authors argue that affective recovery in depression is accompanied by a measurable functional reorganization of brain networks, and qEEG thus becomes a sensitive marker of network changes associated with therapy. Novel antidepressant mechanisms are being characterized within the same pharmacological framework: Sanacora et al. [99] reported that the low-trapping NMDA-channel blocker lanicemine produced sustained antidepressant efficacy with minimal psychotomimetic effects, the type of drug action whose cortical network effects pharmaco-qEEG is increasingly used to track. This ability to detect functional changes has significant potential to guide therapeutic decisions in clinical practice, for example, by early identification of response to a particular antidepressant and by adjusting treatment accordingly. Overall, the three studies illustrate complementary ways in which qEEG may capture the reconfiguration of cortical networks induced by therapeutic interventions: pharmacological (rivastigmine in Parkinson’s dementia), behavioral (neurofeedback in epilepsy) and psychopharmacological (antidepressants in depression). Although none of the studies had as a main objective the direct demonstration of neuroplastic mechanisms, the data provided support the hypothesis that functional changes measurable by qEEG are correlated with clinical development and could serve in the future as biomarkers of the therapeutic response in both human medicine and, by translational extension, in veterinary medicine.

#### 7.2.2. Post-Vascular Rehabilitation and Functional Recovery

Beyond pharmacological interventions and neurofeedback, an area in which qEEG is increasingly clearly demonstrating its usefulness is that of post-vascular rehabilitation. Although not all studies in this field are interventional for the purpose of active rehabilitation, they provide solid evidence that background EEG patterns are closely related to the trajectory of functional and cognitive recovery after stroke.

Song et al. [89] showed, in a longitudinal cohort study in post-stroke patients, that the presence of a slowed background rhythm (dominant frequency below 7.4 Hz) and an increase in spectral power in the theta band at baseline predicted the further development of cognitive deficit. Similarly, Finnigan et al. [90] used qEEG to stratify the risk of post-stroke cognitive deficit, demonstrating that the quantitative parameters, in particular the degree of background slowdown and the ratios between slow and fast bands, are significantly correlated with the subsequent clinical development. Although these studies did not involve a rehabilitation intervention proper, they provide an essential tool for early triage of patients: based on the qEEG signature from the acute moment, the clinician can estimate the recovery potential and allocate rehabilitation resources to the best places to benefit from them. In addition, the same parameters can be re-evaluated during the rehabilitation program, providing an objective measure of progress independent of subjective patient reporting or clinical scales, which can be influenced by psychological or waiting factors.

An even more direct contribution in this field comes from Alwhaibi and collaborators [100], who conducted a randomized controlled trial (RCT) in patients with chronic stroke and lower limb locomotor deficiency. In this robust design, the intervention group benefited from standard physical therapy associated with visual and auditory somatosensory stimulation, while the control group followed only standard physical therapy. The results showed that the intervention group had both a significant increase in functional independence and a qEEG pattern characterized by a “generalized activation of the cortex and an increase in spectral power in the rapid bands (especially beta). The correlation between electrophysiological changes and clinical improvement supports the idea that the effects of rehabilitation are not limited to the peripheral level (muscular, articular), but involves a functional reorganization of cortical networks, a form of neuroplasticity induced by active recovery. This study is therefore a direct demonstration of the ability of qEEG to capture the effect of rehabilitation on brain plasticity, providing an objective biomarker of therapeutic response. Although the study was conducted on human subjects, the implications for the canine model are significant: to the extent that dogs with post-stroke neurological deficits (or posttrauma) are subject to rehabilitation programs, the same qEEG parameters could be used to monitor response and adjust the intensity of intervention in a personalized manner.

#### 7.2.3. Network Plasticity in Development and in Cognitive Disorders

A complementary perspective on neuroplasticity, different from both pharmacological and behavioral interventions, is provided by studies investigating the dynamics of EEG networks under normal development or psychotic disorders. This research does not aim at rehabilitation proper, but they conceptualize functional plasticity as a fundamental property of brain networks, the ability to adaptively reconfigure in time, and demonstrate that qEEG can measure this ability both in physiological and pathological contexts.

Naim-Feil et al. [62] investigated the dynamics of EEG networks in patients with schizophrenia during a sustained attention task (SART—Sustained Attention Response Task). Using advanced metrics derived from graph theory such as clustering coefficient, minimum path length and how they change during the task the authors demonstrated that patients with schizophrenia exhibit reduced functional network flexibility compared to healthy subjects. In other words, while the brain of the patient without schizophrenia quickly reorganizes its connectivity according to the demands of cognitive task, that of the patient with schizophrenia remains in a more-rigid, less-able-to-adjust configuration. Although this study is not one of rehabilitation in the classical sense, the concept it explores is directly applicable to the field: functional neuroplasticity can be understood as exactly this adaptive reconfiguration ability over time, and qEEG provides the tools to measure it. A successful therapeutic intervention, whether pharmacological or behavioral, should be reflected in an increase in this flexibility, a hypothesis that can be tested directly with the same network metrics.

At the opposite pole of the age spectrum, Falivene et al. [101] longitudinally analyzed the development of EEG networks in healthy infants, between the ages of 6 and 12 months, a critical period of cognitive maturation and sensorimotor maturation. The results showed that both coherence (a measure of functional connectivity between regions) and functional integration (the ability of the network to combine information from distinct areas) increased significantly over these six months. More importantly, these electrophysiological changes significantly correlate with performance in standardized neurobehavioral development tests, thereby validating the functional significance of network parameters. The study therefore provides a physiological model of normal neuroplasticity: qEEG maps, in a non-invasive and quantitative way, the maturation of cortical networks during postnatal development.

The significance of this model for the field of rehabilitation and therapy is profound. Once we have a map of the normal trajectory of EEG connectivity in development, we can set objective milestones to evaluate, both in children with developmental delays or in patients with brain damage, the degree to which functional networks approach or move away from this physiological trajectory. In addition, the same metrics can be used to evaluate the response to therapeutic interventions: a successful rehabilitation should be reflected in a normalization of network parameters, that is, in a movement of them to the values observed in typical development. Thus, the studies of Naim-Feil and Falivene, although apparently distant from the central subject of canine epilepsy, provide a conceptual and methodological framework essential for understanding how qEEG can capture functional plasticity at both ends of the age spectrum and under various pathological conditions.

#### 7.2.4. Mechanisms Indexed by the Observed qEEG Changes

The convergence described above is not merely descriptive, and it is worth stating which mechanisms the observed qEEG changes are likely to index, since this determines what a qEEG endpoint can and cannot demonstrate. Alpha activity in the 8–13 Hz range is generated largely by thalamocortical and cortico-cortical loops whose synchrony is modulated by ascending cholinergic and aminergic projections [2,56,57]. The increase in alpha power that follows cholinesterase inhibition in Parkinson’s disease dementia [65] is therefore consistent with restored cholinergic drive to these loops rather than with structural repair, which is why it appears within weeks and why it may dissociate from the degree of cognitive gain. Conversely, an excess of delta and theta activity is the electrophysiological signature of cortical deafferentation and of reduced effective connectivity between regions, and its reduction under treatment is best read as partial restoration of that connectivity rather than as neuronal recovery.

The behavioral interventions act through a different mechanism. In qEEG-guided neurofeedback the contingency between a spectral feature and a reinforcing signal constitutes operant conditioning of a cortical rhythm, and the persistence of the change after the protocol ends [58] is what would be expected of activity-dependent synaptic modification rather than of a pharmacological effect, which reverses on withdrawal. In sensorimotor rehabilitation, the generalized cortical activation and the increase in fast-band power reported after somatosensory training [100] are consistent with the reorganization of sensorimotor representations described in the wider plasticity literature, in which repeated afferent input alters the cortical territory responding to it. Because coherence and graph-theoretical measures quantify how distributed the resulting network becomes, rather than how active any single region is, they are in principle the descriptors best matched to a plasticity endpoint [57,62].

Two consequences follow for the canine literature. First, the spectral redistribution induced by phenobarbital [36] admits two readings, and the canine data do not yet separate them. Phenobarbital acts as a positive allosteric modulator at the GABA-A receptor, prolonging chloride channel opening and hyperpolarizing the membrane, which is the standard mechanism by which barbiturates suppress cortical excitability; by this reading the spectral change is a direct pharmacological effect. The alternative reading is that excess delta activity in a chronically epileptic brain partly reflects the cortical dysfunction produced by recurrent pathological hypersynchrony, so that its reduction under treatment marks a network returning toward a more physiological mode of operation rather than a sedative artifact. The second reading is the more interesting one translationally, but a drug that shifts cortical excitability will alter the spectrum whether or not the network has changed, and distinguishing the two requires that the recording be repeated after a washout or at a stable dose over time, which no canine study has yet done. Second, the frontal alpha increase observed in dogs at risk of cognitive decline [85] is compatible with a compensatory recruitment of frontal networks, but an equally parsimonious reading is early cholinergic dysregulation; only a longitudinal design, in which the same animals are followed as they convert, can separate compensation from prodrome. Framing the canine findings mechanistically therefore makes the required study design explicit rather than merely desirable.

### 7.3. Translational Human–Dog Model in Rehabilitation and Neuroplasticity

#### 7.3.1. Preservation of Mechanisms of Functional Plasticity Between Species

The studies reviewed in this section, summarized in Table 2, converge on a single point: in both species the qEEG changes that accompany disease progression and therapeutic intervention are systematic rather than incidental, and they involve the same descriptors, namely spectral power, coherence and functional connectivity. In human patients this has been demonstrated for cholinergic therapy, for qEEG-guided neurofeedback, for antidepressant treatment and, in a randomized design, for physical rehabilitation [58,65,70,100]. In the dog the corresponding evidence is indirect, resting on the spectral redistribution induced by phenobarbital and on the frontal alpha increase observed in at-risk animals [36,85]. The mechanisms of functional plasticity therefore appear to be conserved between the species, which supports the dog as a model for their study; the important qualification is that no canine study has yet used qEEG as an endpoint of a rehabilitation intervention, so the canine side of this parallel remains inferential.

The preservation of these interspecies mechanisms validates the dog as a natural model for the study of functional plasticity in the context of neurological disease and paves the way for the use of canine qEEG as a biomarker of response to therapeutic interventions, from antiepileptic drugs to cognitive rehabilitation programs.

#### 7.3.2. Implications for Translational Research and Clinical Practice

The translational model emerging from this synthesis has significant implications for both research and clinical practice:

1. Validation of canine models for rehabilitation studies. Existing data suggest that, like humans, the dog exhibits measurable qEEG changes associated with disease progression and response to treatment, which validates the species for preclinical rehabilitation and therapeutic-induced neuroplasticity studies.

2. Development of translational biomarkers of therapeutic response. qEEG parameters that have been shown to be sensitive to human interventions (e.g., spectral power in fast bands, interhemispheric coherence, network metrics) can be prospectively validated in dogs, thus creating a common set of biomarkers applicable in both species.

3. Optimization of rehabilitation protocols. The use of qEEG as a therapeutic guide tool, as proposed by Walker [58] for neurofeedback, can be extended to other interventions (physiotherapy, cognitive stimulation, pharmacological therapies), allowing customization of protocols according to individual electrophysiological profile.

4. Objective monitoring of recovery. qEEG provides a quantitative, objective and non-invasive measure of the reorganization of brain networks during recovery, complementary to clinical and behavioral assessments, both in humans and dogs.

Thus, the field of neurological rehabilitation and neuroplasticity is one of the most promising directions of application of translational qEEG, with the potential to accelerate both the development of new therapeutic interventions and the optimization of existing protocols by using objective biomarkers, which are comparable between species.

## 8. Autonomic and Paroxysmal Disorders

This category brings together conditions characterized by paroxysmal episodes, seizures, parasomnias, and autonomic events, whose nature (epileptic versus nonepileptic) can be elucidated only by EEG registration. Their inclusion in this synthesis is justified by the fact that, although there are not traditionally areas of application of qEEG, they are areas where quantitative analysis can bring major diagnostic benefits, in particular by automatic classification of events and by objective differentiation between epileptic and nonepileptic patterns.

### 8.1. Canine Component—RBD, Autonomic Crises and Paroxysmal Events

In veterinary medicine, three clinical entities illustrate the importance of differential diagnosis based on EEG in paroxysmal disorders. All three have remarkable phenotypic correspondences in human neurology, and their understanding can benefit from the application of quantitative and machine learning techniques.

The first entity is REM sleep behavior disorder (RBD), documented in the dog by Bush et al. [40]. The case study reported a dog with epilepsy and associated neurodegenerative conditions, in which monitoring of polysomnographic EEG confirmed that complex behavioral episodes characterized by extensive movements, vocalizations and disorganized motor behaviors occur exclusively in REM sleep, in the total absence of epileptiform activity. This finding validates canine RBD as a spontaneous model for appropriate human pathology and stresses the importance of differential diagnosis: what can be clinically confused with nocturnal epileptic seizures turns out, on close examination, to be a nonepileptic parasomnia. The use of quantitative analysis of EEG patterns during sleep, for example, quantifying the percentage of REM-specific sawtooth activity or muscle tone changes could in the future enable automatic classification of these events, reducing the need for subjective visual interpretation. The canine RBD phenotype has since been documented in further contexts: Knipe et al. [103] confirmed, by time-locked video EEG, REM-associated motor behaviors without epileptiform activity in a dog with generalized tetanus, again distinguishing a parasomnia from nocturnal seizures. The value of EEG for classifying paroxysmal events extends beyond the dog: Brewińska et al. [104] characterized generalized tonic seizures accompanied by paroxysmal fast activity in a Tonkinese cat, supporting a Tier-III diagnosis of idiopathic epilepsy and illustrating the applicability of the same electrographic criteria across companion-animal species.

The second entity is the focal autonomic crises, described in the dog under various names: panic attacks, episodes of paroxysmal restlessness, recurrent digestive or cardiac manifestations without identifiable peripheral substrate. In the absence of obvious convulsive motor manifestations, the differential diagnosis between autonomic epilepsy and other nonepileptic paroxysmal disorders (e.g., primary dysautonomies, paroxysmal anxiety disorders) is extremely difficult based solely on clinical signs. EEG recording, preferably long-term, including during sleep, can identify subclinical epileptiform discharges or ictal patterns located in regions such as the insular or orbitofrontal cortex, which are associated with autonomic manifestations. Quantitative analysis of spectral power and coherence in the preictal period in dogs is still in its infancy, but the principles drawn from the human literature (for example, the identification of predictive spectral patterns) are directly applicable.

The third category is that of undifferentiated paroxysmal events: sudden, short-term behavioral episodes that may resemble epileptic seizures but may be of a nonepileptic nature (e.g., paroxysmal movement disorders, catalepsy, narcolepsy, or even episodes of acute pain). In dogs, these situations are common in neurological practice, and differential diagnosis is often based on exclusion or response to antiepileptic treatments, a strategy that can delay the correct diagnosis and expose the animal to unnecessary medication. Continuous video EEG, analyzed quantitatively, can provide objective criteria for differentiation: for example, the presence or absence of ictal epileptiform discharges, post-ictal changes in the background rhythm, or specific patterns of EEG activity during the episode.

Although directly applicable human studies in this category are missing from the interrogated databases, methodological and computational principles extracted from the existing literature, in particular the validation of common machine learning platforms [45], pave the way for the development of computer-aided diagnostic tools applicable in both species. For example, an SVM algorithm trained to differentiate between ictal and nonictal EEG patterns in human epilepsy could be tested, with careful standardization, on canine data and if the performance is similar, it would validate both the portability of the algorithm and the phenomenological similarity of events between species. In the absence of such tools, differential diagnosis of paroxysmal disorders in the dog remains a difficult area, where quantitative qEEG has significant potential, which is still insufficiently exploited.

#### 8.1.1. Paroxysmal Events—Epileptic Versus Nonepileptic

A recent and relevant contribution to this category comes from Lyon et al. [98], having directly addressed the clinical problem of paroxysmal events in dogs and cats, a frequent challenge in veterinary practice, where differentiation between epileptic seizures and nonepileptic episodes based solely on clinical signs is often ambiguous. The prospective study aimed to develop and validate a standardized short-term, nonsedated EEG protocol specifically designed to triage paroxysmal events under veterinary conditions.

The results obtained are remarkable from a practical perspective. First, the success rate of unsedated entries proved extremely high (about 94%), indicating that, contrary to some previous perceptions, EEG can be performed in dogs and cats without sedation, with a high degree of feasibility, thus reducing the risks associated with anesthesia and eliminating the confounding effects of sedatives on EEG patterns. Secondly, the protocol allowed them to obtain interpretable EEG data, capable of identifying characteristic epileptiform patterns (spikes, spike–wave complexes, etc.) and, at the opposite pole, to recognize normal EEG activity in nonepileptic events—thus providing an objective criterion for the exclusion of epilepsy. Thirdly, based on these findings, the authors propose a practical algorithm for integrating EEG into the evaluation of paroxysmal events, which can be implemented in the veterinary clinic workflow.

The significance of this study for translational research and future qEEG applications is substantial. By establishing a standardized, feasible and non-invasive protocol, Lyon and collaborators are laying the foundation for a veterinary clinical neurophysiology standard for paroxysmal events. This methodological infrastructure is the precondition for the development of quantitative applications in this area, from the automatic classification of EEG patterns (epileptic versus nonepileptic) using machine learning algorithms, to the identification of differential biomarkers allowing early diagnosis even in the absence of an event during registration. In addition, the fact that the protocol has been validated on two species (dog and cat) suggests that its principles are generalizable, and the success of unsedated records paves the way for longitudinal studies in which animals can be monitored repeatedly, without additional stress and without the confounding effects of sedation.

#### 8.1.2. Focal Autonomic Seizures—“Atypical” Paroxysms with Epileptic Substrate

Another clinical entity that is difficult to diagnose, but essential for understanding the spectrum of paroxysmal disorders in dogs, is represented by autonomic focal seizures. They are predominantly manifested by gastrointestinal or visceral signs, the absence of classical convulsive motor manifestations frequently leading to initial suspicions of primary digestive pathology.

Diop et al. [102] described a series of three dogs with such autonomic focal seizures, manifested by vomiting, epigastric pain and hypersalivation, symptoms that, in the absence of an EEG, would almost certainly have been attributed to a gastroenterological condition. In all three cases, however, the EEG recording revealed focal epileptiform activity, compatible with the diagnosis of autonomic epilepsy. This clinical presentation “deceptive” highlights two fundamental aspects. First of all, autonomic crises can almost perfectly mimic a primary visceral pathology, and differentiation requires a high index of suspicion, and especially electrophysiological confirmation. Secondly, the EEG, even in its interictal variant, without capturing an event in real time, may be sufficient to guide the diagnosis by identifying focal epileptiform discharges in cortical regions associated with autonomic control (for example, the insular cortex or cingulate cortex).

From a translational perspective, Diop’s study [102] and collaborators raise the conceptual level of the discussion. Not all paroxysmal autonomic events are “functional” or of peripheral origin; some have an authentic cortical epileptic substrate. This finding, well established in human neurology (where isolated autonomic seizures are recognized as a form of focal epilepsy), is now also documented in the dog, albeit in a small case series. The EEG remains the only tool capable of confirming the epileptic nature of these events, and the application of quantitative analysis (for example, quantifying the coherence between cortical regions involved in autonomic control or automatic classification of interictal EEG patterns) could further increase the sensitivity and specificity of the diagnosis. In the absence of such an instrument, dogs with focal autonomic seizures risk being subjected to extensive and unnecessary digestive investigations, delaying the establishment of appropriate antiepileptic treatment.

### 8.2. Human Component—Direct Data Absent, Transferable Principles

In the databases analyzed for this review, no human studies dedicated exclusively to autonomic or paroxysmal disorders such as psychogenic nonepileptic crises (PNES), syncope, focal autonomic crises or behavioral disorder in REM sleep (RBD) were included. However, the absence of such studies does not mean that this clinical category is not translational in relevance. On the contrary, methodological and computational principles developed in other contexts can be applied directly in this field.

The most important contribution to this comes from Levitt et al. [45], who developed and validated a machine learning algorithm (Support Vector Machine—SVM) applied to EEG data from humans, dogs and rats. The study demonstrated that the same preprocessing pipelines and the same statistical and qEEG characteristics can be used robustly in humans and dogs, achieving similar performance in the classification between clean signals and a signal contaminated with artifacts.

The relevance of this study for paroxysmal disorders is triple. First, they demonstrate that the same machine learning architecture can work in both species, thus paving the way for the development of classifiers capable of automatically differentiating between epileptic and nonepileptic paroxysmal events, a major diagnostic challenge in both human and veterinary neuroscience. Secondly, the SVM algorithm is particularly suitable for the detection of artifacts in short, unsedated records—exactly the type of records proposed by Lyon et al. [98] for paroxysmal event triage in the veterinary practice. Thirdly, the study validates the concept of common preprocessing and analysis pipeline for human and canine EEG, which means that an algorithm trained on a large set of human data can be applied, after minimal recalibration, to canine data.

Thus, although Levitt and the collaborators [45] have not directly studied the PNES or autonomic seizures, the technology upon which they developed is exactly the necessary foundation for a future automatic diagnosis of paroxysmal disorders in clinical practice in both humans and dogs.

### 8.3. Translational Human–Dog Model for Autonomic and Paroxysmal Disorders

The three canine entities described above correspond closely to entities well characterized in human neurology, and the correspondence is electrophysiological rather than merely descriptive. Canine REM sleep behavior disorder reproduces the defining features of the human parasomnia, including loss of physiological muscle atonia and the absence of epileptiform discharges [40]; canine focal autonomic seizures presenting with gastrointestinal signs parallel the autonomic seizures recognized in Panayiotopoulos syndrome and in mesial temporal epilepsy [102,105]; and the triage problem addressed by the unsedated protocol of Lyon et al. [98], which is the problem faced daily in human epileptology, namely the separation of epileptic seizures from syncope, parasomnias and psychogenic nonepileptic events. These correspondences are summarized in Table 2. In this domain, unusually, the canine phenotypes are better described than the quantitative tools available to classify them, which is why automated discrimination of epileptic from nonepileptic events remains the obvious next step in both species.

Thus, in the dog, paroxysmal phenotypes directly comparable to human ones are described: RBD as paroxysmal parasomnia, focal gastrointestinal autonomic seizures and a wide range of paroxysmal events evaluated by nonsedated EEG. These entities are analogous to human RBD, autonomic vegetative seizures, and differential diagnosis situations between epilepsy, PNES, and syncope. The dog thus emerges as a spontaneous model relevant to the differentiation of epileptic events from nonepileptic ones, offering the unique opportunity to study these disorders under controlled conditions, but preserving the complexity and clinical variability of a natural pattern.

#### Mechanisms Underlying the Cross-Species Correspondence

The correspondences summarized above acquire their translational weight only if the mechanisms generating each phenotype are comparable between the species, and in these three entities they plausibly are. Autonomic seizure semiology is attributed in human epileptology to ictal recruitment of the insular and cingulate cortices, which form the cortical component of the central autonomic network [106]; nausea, emesis, hypersalivation and pallor are the expected output of that recruitment rather than incidental accompaniments. The canine cases reported by Diop et al. [102], in which gastrointestinal signs were accompanied by focal epileptiform discharges, are consistent with the same substrate, and this is what makes the phenotype informative rather than merely curious: it predicts where the abnormality should be found. It also identifies the quantitative target, since coherence between insular, cingulate and adjacent temporal derivations is the measure that would test the hypothesis directly, and it has not yet been computed in either species for this presentation.

REM sleep behavior disorder rests on a different and better-characterized mechanism. The atonia of normal REM sleep depends on brainstem circuitry, and its loss permits the enactment of dream content; in human patients this loss is associated with α-synuclein pathology, and prospective follow-up of a large multicenter cohort has shown that most affected individuals convert to Parkinson’s disease, dementia with Lewy bodies or multiple-system atrophy, at a rate of about 6% per year [107]. The canine case documented by Bush et al. [40] reproduced the defining electrophysiological feature, namely sustained electromyographic activity during REM sleep without epileptiform discharge, in an animal with concurrent neurodegenerative disease. Whether the canine disorder carries the same prognostic implication is unknown, and it is a question that only prospective follow-up of affected dogs can answer; the value of the phenotype for translational research depends on that answer.

The mechanistic reading also explains why this domain is the one in which quantitative analysis is least developed and most needed. Both the differentiation of epileptic from nonepileptic paroxysms and the separation of a parasomnia from a nocturnal seizure are, formally, classification problems on short segments of signal, and they are precisely the problems for which the cross-species pipeline validated for artifact detection [45] provides an existing architecture. What is missing is not the method but the labeled data: the standardized unsedated protocol of Lyon et al. [98] supplies the acquisition conditions under which such a dataset could be assembled, and the canine phenotypes described in this section supply the categories it would have to distinguish.

### 8.4. Dependence on EEG, qEEG Infrastructure and Future Translational Directions

A first common element of all the canine studies analyzed in this category is the fundamental dependence on EEG (and, in the case of RBD, polysomnography) for establishing the correct diagnosis. In all three clinical situations, RBD, focal autonomic crises and undifferentiated paroxysmal events, a single clinical examination proved insufficient. Bush et al. [40] showed that without EEG recording, the dog’s nocturnal behavioral episodes with RBD could have been confused with nocturnal epileptic seizures. Diop et al. [102] demonstrated that paroxysmal gastrointestinal signs, in the absence of EEG, initially led to the suspicion of a primary digestive pathology, delaying the correct diagnosis of autonomic epilepsy and Lyon et al. [98] validated an unsedated EEG protocol precisely to provide an objective triage tool in first-line practice, where differentiation between epileptic seizures and nonepileptic events is impossible based solely on clinical signs. This dependence on EEG is perfectly similar to that in human medicine, where polysomnography is the gold standard for the diagnosis of RBD, and video EEG is mandatory for the differentiation of epileptic seizures from psychogenic nonepileptic seizures (PNES) or syncope.

Secondly, although qEEG is not yet extensively used in these pathologies in the dog, the infrastructure necessary for its implementation has already been established. Lyon et al. [98] have developed a reproducible protocol for the acquisition of nonsedated EEG, with clear technical criteria (electrode placement, recording duration, environmental conditions), aligned to the existing recommendations for the human EEG. Levitt et al. [45] demonstrated that the same statistical characteristics and the same SVM algorithm can be successfully applied to human and canine EEG for automatic classification, a proof of concept that common metrics are feasible. In addition, canine studies have already begun to characterize distinct EEG patterns for different types of paroxysmal events: REM patterns without atony in RBD, focal epileptiform discharges in autonomic seizures, and normal EEG activity in nonepileptic events. These patterns can serve as “ground truth” for training and validating future machine learning algorithms.

Thirdly, by validating an SVM architecture applicable to both human EEG and canine EEG, it becomes feasible to develop a common classification model integrating data from both species. Human data EEG patterns from epilepsy, PNES, syncope and RBD can be combined with canine data patterns from idiopathic epilepsy, canine RBD, focal autonomic seizures and nonepileptic paroxysmal events. Such a model could be trained to discriminate between epileptic versus nonepileptic events, between autonomic epileptic seizures versus primary autonomic dysfunctions, or between parasomnias (RBDs) versus nocturnal seizures. Clinical applications would be multiple: in veterinary practice, a computer-aided diagnostic tool could provide a quick and objective decision during a short-term EEG registration; in translational research, the same model could serve to validate common biomarkers for preclinical studies of paroxysmal disorders. Thus, the dog is no longer just a spontaneous model for isolated clinical phenotypes, but becomes an active partner in the construction of a unified computational framework, able to accelerate the transfer of discoveries from the laboratory to clinical practice, both in humans and animals.

## 9. Conclusions and Future Directions

Before the limitations of this synthesis are set out, the evidence reviewed in the six thematic sections is consolidated in Table 2, which places the human and the canine findings of each domain side by side and states the gap that separates them. Read across its rows, the table makes the asymmetry of the field explicit: the two species converge closely in epilepsy and in cognitive decline, diverge in the methodological domain, where the canine standard remains to be established, and are entirely unmatched in acute brain injury.

### 9.1. Limitations of the Evidence and of This Review

Several limitations should be considered when interpreting this synthesis. First, the included body of evidence was methodologically heterogeneous in design, recording montage, state of consciousness and analytical pipeline, which precluded any quantitative synthesis or meta-analysis and confined the review to a narrative, comparative approach. Second, a formal, standardized risk-of-bias assessment was not applied; study quality was appraised only narratively, and the review protocol was not prospectively registered. Third, the canine evidence base is small and uneven: several key observations rest on single case reports or pilot studies, longitudinal and multicenter veterinary data are largely absent, and whole diagnostic areas, most notably acute brain injury, are represented in the dog only by extrapolation from human work (Figure 2A); moreover, almost half of the canine studies retained here were published after 2020 (Figure 2B), so a substantial part of this evidence has yet to be independently replicated. Fourth, the biomarker placed at the center of this review, EEG coherence, is at present far better supported in humans than in dogs, where canine coherence data derive essentially from a conditioning study and a methodological electrode comparison, with no study yet characterizing coherence in canine epilepsy proper; the cross-species claims for coherence should therefore be read as hypotheses to be tested rather than as established equivalences. Fifth, the cross-species machine-learning evidence, although encouraging, currently concerns automated artifact detection rather than the classification of disease states, so its translational reach remains primarily methodological. Sixth, anesthesia and sedation (almost unavoidable in canine recordings) together with chronic antiepileptic treatment, constitute major confounders that were documented unevenly across the included studies and were not formally tabulated. Finally, as a single-team integrative review the literature-search and selection process is subject to selection and language limitations, and the number of included studies should be regarded as the corpus that met the eligibility criteria rather than an exhaustive census of the field. These constraints temper the strength of the conclusions and define the priorities, standardized acquisition protocols, normative canine databases, and prospective validation, outlined below.

### 9.2. The Need for Longitudinal Studies in Naturally Occurring Canine Disease

One structural limitation runs through the whole of the veterinary literature reviewed here: longitudinal designs are rare. The scale of the problem is visible even in the largest canine cohort assembled to date. The Dog Aging Project reported cognitive assessments on 15,019 companion dogs, yet the analysis rested on first-year enrolment data and was explicitly cross-sectional rather than a follow-up in time [108]. If a sample of that size remains a photograph rather than a trajectory, the uncertainty is greater still in the much smaller datasets typical of the canine qEEG literature, where the absence of time-tracked cohorts makes it impossible to distinguish a stable biomarker of the disease from a transient fluctuation without prognostic value.

That canine neurological disease arises spontaneously rather than being experimentally induced is precisely what gives it translational value [24], but the same property makes longitudinal data harder to collect than in a laboratory model, where the moment of onset is known and controlled. The obstacle is nevertheless surmountable, and the infrastructure for doing so already exists: a longitudinal program within the Dog Aging Project has been established specifically to follow companion dogs annually for brain and cognitive health, with biofluid banking and post-mortem examination [109]. A comparable design adapted to qEEG, with recordings repeated at standardized intervals in the same animals followed from a preclinical stage to manifest disease, would convert qEEG from a diagnostic measurement at a single time point into a biomarker of progression, which is the role it already occupies in human neurology.

### 9.3. Concluding Remarks

The present literature review demonstrates that quantitative electroencephalography (qEEG) is a robust methodological bridge between human and canine research, with applications extending from epilepsy (the best explored field) to acute brain injury, neurodegeneration, neurological rehabilitation and paroxysmal disorders. The comparative analysis of the 75 studies included reveals a remarkable convergence of analysis principles, quantitative biomarkers and methodological frameworks between the two species, supported by the demonstration that the same computational platforms and machine learning algorithms can be applied with similar performance on human and canine EEGs.

In the field of epilepsy, the dog has strengthened its position as a natural model of human focal epilepsy, with EEG patterns and pharmacodynamic responses to antiepileptic treatment remarkably similar to those described in humans. In neurodegeneration, canine cognitive dysfunction syndrome (CCD) emerges as a spontaneous model of dementia, with changes in qEEG and sleep architecture closely mirroring those described in Alzheimer’s disease and in the early stages of human cognitive decline. In acute brain injury, although canine studies are lacking, the methodological principles established in the human literature—anchoring in normative databases, separation of pharmacological effects, validation of cross-species biomarkers—provide a ready-to-apply framework for the development of this field in veterinary medicine. In rehabilitation and neuroplasticity, qEEG is a sensitive marker of dynamic reorganization of cortical networks induced by therapeutic interventions, with applications extending from neurofeedback to physiotherapy and pharmacological treatment. In autonomic and paroxysmal disorders, the description of canine RBD, focal autonomic seizures and standardized protocols of unsedated EEG pave the way for the development of tools for the automatic classification of epileptic versus nonepileptic events, applicable in both species.

However, this synthesis also exposes an uneven distribution of evidence between the two species. The human literature rests on decades of conceptual consolidation, large normative cohorts and clinically implemented applications; the canine literature, by contrast, remains fragmented and insufficiently standardized. Canine epilepsy is well characterized, but acute brain injury has yet to receive any dedicated electrophysiological study; there are promising results in CCD, but age-related norms and longitudinal cohorts are lacking; acquisition protocols are beginning to converge, yet the influence of pharmacological factors and electrode type on quantitative parameters is still poorly documented.

These gaps should be read less as deficiencies than as a research agenda, and the specific directions arising from each domain have been detailed in the corresponding partial conclusions (Section 4). What they share is a common precondition: none can be met at the scale of a single institution. The temporal profile of the present corpus (Figure 2B) indicates why these directions are best pursued collectively rather than center by center. Because the canine evidence is recent and rests on small, breed-restricted samples, single-center studies are unlikely to reach the sample sizes required for age- and breed-stratified normative data. Multicenter collaboration between veterinary teaching hospitals and referral institutions, using the common acquisition and reporting standard proposed in Table 1, would allow recordings to be pooled prospectively, and longitudinal follow-up of naturally occurring canine disease would supply the repeated-measures data that cross-sectional designs cannot provide. Such pooling also depends on expert-annotated reference datasets: the cross-species classifier of Levitt et al. [45] performed well precisely because it was trained against expert-labeled recordings, and any extension of machine learning from artifact detection to disease-state classification will require canine ground-truth annotation of comparable quality.

Two features of veterinary neurology make such collaboration more than an aspiration. Standardization is its precondition and also its first product: uniform recording and analysis protocols, of the kind proposed in Table 1, are what would make records from different institutions comparable at all, and agreeing to them is the least expensive part of the exercise. Epilepsy is the natural place to begin, because it is common, because it permits repeated recording over the clinical course, and because long-term anticonvulsant therapy provides the pharmacological contrast against which spectral change can be interpreted. The experience gained there could then be extended to the other conditions in which functional change is diagnostically relevant, and it is worth saying plainly that this is where the field would have to go if qEEG is to become a clinical instrument in veterinary neurology rather than a research one.

Ultimately, what unites all these areas is the very nature of qEEG: a quantitative, objective and non-invasive window into brain function that transcends specific barriers and allows for direct comparison of physiological and pathological mechanisms between species. As veterinary medicine adopts the already established methodological standards in human neurology, and translational research will increasingly integrate natural models such as a dog with spontaneous epilepsy or cognitive dysfunction, we will witness the consolidation of a common language of clinical electrophysiology with mutual benefits for human and animal health. This synthesis is a step in this direction, providing both a map of what has already been achieved and a guide to what remains to be built.

## Figures and Tables

**Table 1:** Proposed minimum specification for the acquisition and reporting of canine qEEG, adapted from the human clinical qEEG standard and from the joint IFCN–ILAE minimum recording standards, and constrained by the canine methodological evidence reviewed in Section 6. The left column lists the element to be specified, the center column the proposed minimum, and the right column the evidence on which the recommendation rests together with the species-specific caveat that applies.

**Element**	**Proposed Minimum for Canine qEEG**	**Basis and Canine-Specific Caveat**
Patient preparation	Fasting interval before sedation where sedation is planned; a quiet room with controlled temperature and minimal external stimulation; the interval since the last antiepileptic dose recorded.	Preparation conditions are almost never reported in the canine studies reviewed here, yet ambient stimulation and arousal alter the background rhythm, and the time since the last antiepileptic dose determines the pharmacological state at which the record is obtained [97].
Electrode array	A canine adaptation of the international 10–20 system, bilaterally symmetric and covering frontal, temporal and occipital regions, with the complete derivation list, the reference and the ground electrode reported.	The human minimum is 19 positions of the 10–20 system [14], and the joint IFCN–ILAE standard recommends the 25-electrode array where possible [97]. Canine arrays remain heterogeneous and the available configurations have been reviewed [23], so the array cannot be assumed from the montage name alone.
Electrode type	Reported explicitly and held constant throughout a study; subdermal needle and surface disk electrodes should not be treated as interchangeable for connectivity analysis.	Electrode type significantly alters interhemispheric coherence values in vigil dogs [44]. This is the single canine-specific constraint with no human equivalent.
State of consciousness	Awake, unsedated recording wherever feasible; where sedation is unavoidable, a sedation–awakening protocol in preference to sedation alone; the state (awake, drowsy, sedated, asleep) documented for every analyzed epoch.	Unsedated recording succeeded in about 94% of dogs and cats under a standardized protocol [98]; sedation–awakening protocols increase the yield of epileptiform discharges relative to sedation alone [31]. Drowsy segments should be excluded from resting-state analysis [14].
Sedation and anesthesia	Where unavoidable, agent, dose, route and interval to recording reported, and the protocol treated as an analysis covariate rather than as background noise.	Medetomidine slows the canine EEG in a dose-dependent manner [30]. In sedated pediatric patients fewer electrographic seizures are detected [78], so a negative sedated recording does not exclude epileptiform activity in the awake state.
Concomitant medication	Antiepileptic and psychoactive drugs reported with dose and, where available, serum concentration at the time of recording.	Phenobarbital shifts the canine relative power spectrum [36], and drug-specific pharmaco-qEEG signatures are established in human patients [53]. Without this information a pharmacological effect cannot be separated from disease progression.
Recording and analysis length	A total recording of at least 20–30 min, yielding 2–5 min of artifact-free signal per analyzed condition, with the duration actually achieved reported rather than assumed.	The human routine record lasts at least 20 min [97] and yields 2–5 min of clean EEG per condition for quantitative analysis, with no retained segment shorter than 1 s [14]. Canine recordings are typically shorter, which makes explicit reporting of the achieved duration essential.
Activation procedures	Where feasible, photic stimulation and a period of natural or drug-free sleep, each reported separately from the resting record.	Activation procedures form part of the human minimum standard and increase the diagnostic yield of a routine record [97]; sleep in particular raises the detection of epileptiform discharges. They are seldom attempted in dogs, and reporting them separately is a precondition for comparing yields between species.
Filter and sampling settings	Sampling rate, band-pass and notch settings reported; the quantitative analysis band stated numerically.	Human clinical qEEG is typically analyzed over approximately 1–40 Hz [14]. Comparability across laboratories fails silently when these settings are omitted.
Artifact handling	Electrode impedance checked before and after the recording; rejection criteria pre-specified; automated detection validated against expert annotation and confirmed by visual inspection; the proportion of the record rejected reported.	Impedance control is part of the human minimum standard [97]. Automated methods require vetting against visual review to avoid both over- and under-rejection [14]; a cross-species classifier has been validated for artifact detection in human, canine and rodent recordings [45], but only against expert-labeled data. In the non-cooperative veterinary patient, movement and muscle artifact are the principal cause of data loss, so the rejected proportion is itself a quality indicator.
Spectral metrics	Band limits defined numerically; absolute power, relative power and symmetry reported; transform and epoch length stated.	Epoch length determines the frequency resolution of the transform, so it is a reporting requirement rather than a technical detail [14]. Reporting both absolute and relative power permits comparison with the human literature [59].
Connectivity metrics	The coherence estimator and the reference scheme reported, and the values interpreted in light of volume conduction and common sources.	Coherence between two derivations may reflect a shared source rather than communication between regions [57]. In the dog this caution is compounded by the electrode-type effect [44].
Normative reference	Deviations expressed as age-stratified z-scores once canine normative data permit, with the reference dataset named.	Z-score referencing against age-regressed norms is the common language of human qEEG [3]. Canine developmental normative data currently derive from 72 dogs recorded under xylazine sedation [68], which limits their applicability to unsedated recordings.
Subject characteristics	Species, breed, skull conformation, age, sex and body weight reported for every animal, together with the acquisition and analysis software used.	This minimum set is systematically absent from the studies reviewed here, and breed-stratified norms and any future pooling of data across centers depend on these variables being available [20,68].

**Table 2:** Cross-species synthesis of the evidence reviewed in Section 3, Section 4, Section 5, Section 6, Section 7 and Section 8. For each thematic domain the table summarizes the human and canine evidence, the principal gap that separates them, and the resulting translational status. Reference numbers indicate the studies discussed in the corresponding section.

**Domain**	**Human Evidence**	**Canine Evidence**	**Principal Gap and Translational Status**
Epilepsy (Section 3)	Interictal spectral and coherence abnormalities detectable without visible epileptiform discharges [11,13,15]; drug-specific pharmaco-qEEG signatures [53]; increased coherence associated with pharmacoresistance [50].	Interictal patterns morphologically equivalent to human focal epilepsy [34]; background qEEG discriminates epileptic from healthy dogs [35]; phenobarbital shifts the relative power spectrum in parallel with seizure control [36].	No study has yet characterized coherence in canine epilepsy itself. Established for spectral power; hypothesis-generating for coherence.
Acute brain injury and neurocritical care (Section 4)	Decline of the alpha/delta ratio predicts delayed cerebral ischemia [54]; qEEG assists triage in mild traumatic brain injury [55]; epileptiform activity is frequent in acute injury [76].	No canine qEEG study was identified in this domain.	The domain is unstudied in the dog. The human analytical framework is transferable, but canine data are absent altogether.
Neurodegeneration and cognition (Section 5)	State-dependent qEEG markers correlate with severity in Alzheimer’s disease [59]; power and coherence discriminate patients from controls [22]; change is gradual across the cognitive continuum [87].	Distinct qEEG signatures in at-risk dogs and in canine cognitive dysfunction, correlated with cognitive test scores [85]; sleep-architecture and spectral changes track severity [86].	Longitudinal cohorts and breed-stratified norms are lacking. After epilepsy, the strongest canine parallel in this review.
Methodology and technology (Section 6)	Minimum technical requirements defined for clinical qEEG [14]; reproducibility and z-score referencing established [3]; interpretative framework available for coherence [57].	Electrode type alters coherence [44]; sedation alters the spectrum dose-dependently [30]; an unsedated protocol has been validated [98]; developmental normative data exist under sedation [68].	No consolidated canine good-practice guideline exists. Table 1 proposes a minimum specification derived from the evidence above.
Rehabilitation and neuroplasticity (Section 7)	Cholinergic therapy increases alpha power in parallel with cognitive improvement [65]; qEEG-guided neurofeedback reduces seizure frequency [58]; somatosensory training alters cortical activation in a randomized trial [100].	Treatment-associated spectral reorganization inferred from pharmacological studies [36]; possible compensatory frontal alpha increase in at-risk dogs [85].	No canine rehabilitation study with qEEG endpoints has been performed. Conceptually open, empirically untouched.
Autonomic and paroxysmal disorders (Section 8)	Autonomic seizures and psychogenic nonepileptic events are well characterized clinically, but no qEEG-specific human study met the eligibility criteria.	REM sleep behavior disorder documented polysomnographically [40]; focal autonomic seizures presenting with gastrointestinal signs [102]; unsedated triage protocol validated in dogs and cats [98].	Automated discrimination of epileptic from nonepileptic events has not been attempted in either species. Here the canine phenotypes are ahead of the quantitative tools.

## Data Availability

No new data were created or analyzed in this study. Data sharing is not applicable to this article.

## Supplementary Materials

The following supporting information can be downloaded at: https://www.mdpi.com/article/10.3390/vetsci13080803/s1, Table S1, Database of translational qEEG studies in canine and human epilepsy included in this integrative review (N = 75 studies, 1994–2025).
